# Three‐dimensional motion corrected sensitivity encoding reconstruction for multi‐shot multi‐slice MRI: Application to neonatal brain imaging

**DOI:** 10.1002/mrm.26796

**Published:** 2017-06-19

**Authors:** Lucilio Cordero‐Grande, Emer J. Hughes, Jana Hutter, Anthony N. Price, Joseph V. Hajnal

**Affiliations:** ^1^ Centre for the Developing Brain and Department of Biomedical Engineering Division of Imaging Sciences and Biomedical Engineering, King's College London, King's Health Partners, St. Thomas' Hospital London UK

**Keywords:** magnetic resonance, image reconstruction, motion correction, neonatal brain imaging, multi‐shot multi‐slice images

## Abstract

**Purpose:**

To introduce a methodology for the reconstruction of multi‐shot, multi‐slice magnetic resonance imaging able to cope with both within‐plane and through‐plane rigid motion and to describe its application in structural brain imaging.

**Theory and Methods:**

The method alternates between motion estimation and reconstruction using a common objective function for both. Estimates of three‐dimensional motion states for each shot and slice are gradually refined by improving on the fit of current reconstructions to the partial k‐space information from multiple coils. Overlapped slices and super‐resolution allow recovery of through‐plane motion and outlier rejection discards artifacted shots. The method is applied to *T*_2_ and *T*_1_ brain scans acquired in different views.

**Results:**

The procedure has greatly diminished artifacts in a database of 1883 neonatal image volumes, as assessed by image quality metrics and visual inspection. Examples showing the ability to correct for motion and robustness against damaged shots are provided. Combination of motion corrected reconstructions for different views has shown further artifact suppression and resolution recovery.

**Conclusion:**

The proposed method addresses the problem of rigid motion in multi‐shot multi‐slice anatomical brain scans. Tests on a large collection of potentially corrupted datasets have shown a remarkable image quality improvement. Magn Reson Med 79:1365–1376, 2018. © 2017 The Authors Magnetic Resonance in Medicine published by Wiley Periodicals, Inc. on behalf of International Society for Magnetic Resonance in Medicine. This is an open access article under the terms of the Creative Commons Attribution License, which permits use, distribution and reproduction in any medium, provided the original work is properly cited.

## INTRODUCTION

Common magnetic resonance imaging (MRI) acquisition protocols take seconds to minutes to complete so that the image quality is vulnerable to subject motion during the scan. Strategies for robustness against motion can be adopted at different levels. First, when possible, motion occurrence can be prevented or limited at the time of scanning. Second, sequence design can be made as tolerant as possible against motion disturbance, which should be made without compromising scan efficiency. Third, remaining artifacts can be reduced and sampled information can be optimally integrated when reconstructing the data.

As for brain imaging, although non‐rigid motion 1 can occur due to pulsatile motion 2 and localized movements can arise such as from eyeball motion 3, the current clinical paradigm relies on the subjects holding their head still enough for the duration of the acquisition to avoid gross motion artefacts. However, in many brain studies, such as for those subjects who have difficulty remaining still enough 4 and in ultra‐high resolution imaging applications 5, large or small motion inconsistencies become a limiting factor, so that appropriate acquisition or reconstruction strategies have to be put in place.

Usual sequences in structural brain studies are based on multi‐shot methods, where a fraction of the k‐space is acquired after a single radio frequency excitation or shot. This type of sampling, particularly when combined with slice selective excitation, provides flexibility to achieve the desired contrast while simultaneously balancing the signal to noise ratio, image resolution, and scanning time requirements. A common operating regime is to use an interleaved scheme in which several slices are sequentially excited within a given repeat time (*T*_R_), that way permitting efficient sampling for large *T*_R_'s 6. In this setting, changes in the head position among different shots and slices, which may be acquired very distant in time, will provoke degradation of individual slices and inconsistencies in volumetric information.

Head motion estimation and/or retrospective motion‐compensated reconstruction in multi‐shot sequences has been extensively studied in the past 7, 8, 9, 10, 11, 12, 13, 14, 15. However, to the best of our knowledge, none of the proposed reconstruction methods can be used to correct for through‐plane motion in multi‐slice acquisitions 1, 3. On the other hand, image‐based alignment methods have been introduced to assemble the information coming from snapshot acquisitions, where individual slices are acquired fast enough to approximately freeze movement, into self‐consistent three‐dimensional (3D) representations of the imaged structures. This has been performed either by matching the structures along slice intersections 16, 17 or by slice to volume registration 18, 19, 20.

In Ref. 15, we proposed a framework for rigid body motion estimation and motion‐compensated reconstruction in volumetric multi‐shot sequences using parallel imaging that is grounded on the sensitivity encoding (SENSE) reconstruction paradigm 21. The method is fully data driven, using a common functional to estimate the motion and the image. Moreover, it is suitable for a wide range of settings as it does not require external sensors, navigators, or particular samplings. In that work, we provided an empirical characterization of the conditions for which, in the absence of noise, fully rigid motion corrected reconstructions are still possible in terms of the amount of motion, number of shots, encoding trajectories, and availability of prior information. Here, the framework is extended to multi‐slice sequences, thus fusing the multi‐shot and aligned snapshot families of motion compensation methods. Moreover, differently from most image‐based alignment methods, our procedure aims to correct for within‐plane and moderate through‐plane motion requiring only a single slice orientation. To support this aim, on the acquisition side, slices are sampled in an overlapped manner (where the slice separation is maintained while the slice thickness is increased for same total acquisition time). On the reconstruction side, first, a motion corrected super‐resolution technique is used to recover from through‐plane motion while considering slice blurring. Second, an outlier rejection strategy is introduced to discard those shots for which the basic assumptions of our framework are not satisfied. Finally, the information from different receiver coils in a head array is used to detect magnetic inconsistencies, to estimate rigid motion, and to interpolate the corrupted spectral information of outlier shots. Once slice and volumetric consistency is improved this way, if two or more orthogonal stacks are available, the result could be connected with a slice to volume reconstruction procedure to obtain 3D nearly isotropic representations of multi‐shot multi‐slice multi‐view datasets. The proposed methodology is applied to the problem of motion correction in *T*_2_‐ and inversion recovery *T*_1_‐weighted fast spin echo scans. The approach is tested in a collection of 517 examinations of neonates studied in natural sleep. Motion correction performance is quantified using two complementary image quality metrics and the ability to correct for inter‐shot and inter‐slice motion, both in‐plane and through‐plane, as well as for artifacted shots is visually illustrated. The source code of a MATLAB implementation of the reconstruction method and the required data to reproduce the results in Figures 3 and 4 of “Visual Validation” section is made available at https://github.com/mriphysics/multiSliceAlignedSENSE/releases/tag/1.0.1.

## THEORY

### Rigid Motion Corrected Multi‐Shot Reconstruction

As introduced in Ref. 15, assuming noise pre‐whitening, the reconstruction with rigid motion correction for parallel multi‐shot volumetric imaging can be formulated in matrix form as:

(1) $$(\hat{x},\hat{T})=\underset{x,T}{\text{argmin}}||AFSTx-y|{|}_{2}^{2},$$

where **y** denotes the measured data, **x** the image to be reconstructed, **T** a rigid motion transformation, **S** a coil sensitivity operator, F a discrete Fourier transform (DFT), and **A** a sampling mask. This formulation is used to reconstruct a 3D image of size $N=N_{1}N_{2}N_{3}$ with *N_i_* the number of voxels along dimension *i* using a coil array of *C* elements from *M* = *ESC* samples of a discretized k‐space grid of size $K=K_{1}K_{2}K_{3}$ where *E* denotes the number of samples per shot and *S* is the number of shots. Detailed information about the terms involved in Equation (1) can be found in Ref. 15, here, we just give a brief description of the structure of the matrices involved:

- **y** is a vector of size M×1.
- **A** is comprised of *SC* × *SC* submatrices of size *E* × *K* that take the value 1 if the sample *e* of the shot *s* corresponds to the sampled k‐space location indexed by *k* and 0 otherwise.
- F is comprised of *SC* × *SC* submatrices of size *K* × *N* corresponding to the 3D DFT with applied k‐space oversampling or downsampling.
- **S** is comprised of *SC* × *S* diagonal submatrices of size *N* × *N* whose diagonal elements correspond to the spatial profile of a given coil *c*.
- **T** is comprised of S×1 unitary submatrices of size *N* × *N* corresponding to the 3D rigid transformation the underlying structure has been subject to when acquiring the shot *s*. A convolution‐based interpolation technique 22 is used to build this matrices, so that the acquired resolution is fully preserved.
- **x** is a vector of size N×1.

### Rigid Motion Corrected Multi‐Shot Multi‐Slice Reconstruction

The extension of the model in Equation (1) to multi‐shot multi‐slice motion correction in the context of both conventional slice selective excitations as well as simultaneous multi‐slice or multiband MRI 23 can be generically formulated as:

(2) $$(\hat{x},\hat{T})=\underset{x,T}{\text{argmin}}||A^{\left(s\right)}FA^{\left(r\right)}HSTx-y|{|}_{2}^{2}+\lambda ||Wx|{|}_{2}^{2},$$

where some new terms have been introduced: **H**, a slice profile filter, **W**, a stabilizer of the reconstruction, and *λ*, a parameter that weights the degree of stabilization required. The sampling matrix **A** has been split into a spectral part, $A^{\left(s\right)}$, and a spatial one, $A^{\left(r\right)}$. In this case, assuming that the slices are arranged along the third dimension, *i* = 3, we want to reconstruct a 3D image from *M* = *ESRC* samples of a discretized hybrid k‐space grid of size $KR=K_{1}K_{2}K_{3}R$, where now *S* is the number of shots per simultaneously excited slices, R=P/Q is the number of excitations to cover the field of view (FOV) in the slice direction, with *P* the total number of excited slices and *Q* the number of simultaneously excited slices or multiband factor, and *K*_3_ corresponds to the blipping pattern cycling period 23. The structure of those terms involved in Equation (2) that differ from their counterparts in Equation (1) is as follows:

- $A^{\left(s\right)}$ is comprised of *SRC* × *SRC* submatrices of size *E* × *K* that take the value 1 if the sample *e* of the shot *s* corresponds to the sampled k‐space location indexed by *k* and 0 otherwise.
- F is comprised of *SRC* × *SRC* submatrices of size $K\times N_{1}N_{2}Q$ corresponding to the 3D DFT with applied k‐space resampling and multiband blipping pattern.
- $A^{\left(r\right)}$ is comprised of $N_{1}N_{2}SRC\times N_{1}N_{2}SRC$ submatrices of size *Q* × *P* that take the value 1 if the slice indexed by *q* in the excitation *r* corresponds to the slice *p* and 0 otherwise.
- **H** is comprised of $N_{1}N_{2}SRC\times N_{1}N_{2}SRC$ submatrices $\tilde{H}$ of size $P\times N_{3}$ that account for the slice profile filter and are further discussed below.
- **W** is comprised of $N_{1}N_{2}\times N_{1}N_{2}$ submatrices $\tilde{W}$ of size $N_{3}\times N_{3}$ that account for the reconstruction stabilization to be applied in the slice direction and are further discussed below.

#### Slice Profile Filter

A signal processing model for multi‐slice acquisitions was proposed in Ref. 24. It assumes that the underlying continuous object has first been convolved by a slice profile and then it has been sampled at some slice locations. The effect of the slice profile is usually that of a low pass filter on the slice direction whereas the sampling period (i.e., slice distance) is inversely related to the spectral overlap. In our reconstruction technique, we will assume that the reconstructed resolution is dense enough so as to neglect the spectral overlapping of those harmonics above the Nyquist limit at that resolution. Then, the submatrices $\tilde{H}$ represent a convolution filter and a spectral overlapping from the reconstructed to the acquired grid, so they can be diagonalized as $\tilde{H}=F_{N_{3}\to P}^{H}{\tilde{H}}^{D}F_{N_{3}\to N_{3}}$, with ${\tilde{H}}^{D}$ a diagonal matrix of size $N_{3}\times N_{3}$ whose diagonal elements represent the slice profile filter, $F_{N_{3}\to N_{3}}$ the 1D DFT at the reconstructed resolution and $F_{N_{3}\to P}^{H}$ the inverse 1D DFT with applied resampling from reconstructed to acquired slice resolution.^1^

#### Reconstruction Stabilizer

In Equation (2), we have chosen a stabilizer based on the $\ell^{2}$‐norm. This is justified by the large computational demands involved in our procedure, so that a closed‐form solution for **x** becomes highly desirable (see “Problem Solving” section below). The role of the stabilizer is twofold. On the one hand, it aids the treatment of the ill‐conditioned deconvolution of the high spatial frequencies that were attenuated by the slice profile filter, that is, it underpins a certain degree of super‐resolution. On the other hand, in the presence of through‐plane motion, the sampling density in the slice direction can no longer be assumed homogeneous so that supporting a certain degree of slice interpolation appears necessary. This motivated the selection of a second‐order finite difference regularizer for this term, as its larger support as compared to the first‐order counterpart was observed to help in slice interpolation.

We should remark that the problem formulation in Equation (2) remains intractable due to the huge size of the matrices involved. Thus, certain term rearrangements and approximations are required to reduce the computational burden. These will be described in the “Algorithmic Implementation” section.

### Problem Solving

The sampling structure of our problem can be described with a compound matrix **E**:

(3) $$E=A^{\left(s\right)}FA^{\left(r\right)}HS.$$

The joint problem in Equation (2) may be addressed in an alternating fashion by iteratively solving the following sub‐problems:

(4) $$\begin{array}{c} \hat{x}=\underset{x}{\text{argmin}}||E\hat{T}x-y|{|}_{2}^{2}+\lambda ||Wx|{|}_{2}^{2}\\ \hat{T}=\underset{T}{\text{argmin}}||ET\hat{x}-y|{|}_{2}^{2}. \end{array}$$

The first sub‐problem admits a closed form solution,

(5) $$\hat{x}={\left(T^{H}E^{H}ET+\lambda W^{H}W\right)}^{-1}T^{H}E^{H}y,$$

which can be computed using the conjugate gradient algorithm.

The solution of the second sub‐problem must satisfy:

(6) $$\nabla^{\left(z\right)}||E\hat{T}(z)x-y|{|}_{2}^{2}=0,$$

with **z** representing the set of translation and rotation parameters of the transformations involved. The solution can be obtained separately for each excitation and shot,

(7) $$\nabla^{\left(z_{rs}\right)}||E_{rs}{\hat{T}}_{rs}(z_{rs})x-y_{rs}|{|}_{2}^{2}=0,$$

where $z_{rs}$ is a 6‐component vector describing the rigid transformation for excitation *r* and shot *s*. We have resorted to the Newton's method to solve this system of equations. The expressions for the gradient and Hessian can be consulted in Ref. 15.

**Figure 1:**
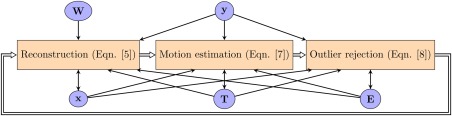
Block diagram of the alternating minimization approach with dynamic outlier rejection.

An outlier rejection criterion is introduced to improve the robustness of the reconstruction against the violation of the model assumptions (either provoked by within‐shot motion, spin history, physiological motion, or other effects). Thus, a feature for outlier rejection is built using

(8) $$t_{rs}=\frac{2||E_{rs}T_{rs}x-y_{rs}|{|}_{2}^{2}}{\sum_{r\prime :P\prime (r\prime )\cap P_{\left\{-1,+1\right\}}(r)\neq ø}\left|\right|E_{r\prime s}T_{r\prime s}x-y_{r\prime s}|{|}_{2}^{2}},$$

where P(r) denotes the set of slices P corresponding to excitation *r* and $P_{\left\{-1,+1\right\}}(r)=P_{-1}(r)\cup P_{+1}(r)$, with $P_{j}(r)$ the set of slices corresponding to excitation *r* with their indexes displaced by *j*. Therefore, this outlier rejection feature is based on the similar nature of the information contained in a given shot for adjacent ( j={−1,+1}) slices. On this basis, a given shot is rejected if $t_{rs}>\tau$. In our implementation, outlier detection is performed at each iteration just after the motion estimation step. Then, shots classified as outliers are removed in the reconstruction step at next iteration (by making $A_{rs}^{\left(s\right)}=0$). The information from these artifacted shots is retrieved by interpolation of neighboring spectral information using the spatial encoding of the coil array and the spatial information from adjacent slices. However, motion estimation is still performed for outliers in subsequent iterations and if it turns that the new motion estimation is able to explain the measured information and, consequently, the error drops below the outlier detection threshold, the data is reincorporated into the information used in the reconstruction. The joint procedure is sketched in Figure 1.

## METHODS

### Acquired Database and Sequence Design

The algorithm was tested on data from examinations on 517 babies (gestational ages at scan ranging from 32 to 45 weeks) who were all imaged in natural sleep using a 3 T Philips Achieva TX with a dedicated *C* = 32‐channel neonatal head coil (RAPID Biomedical) and subject handling system 25. *T*_2_‐ and inversion recovery *T*_1_‐weighted multi‐shot multi‐slice fast spin echo sequences were acquired for each subject in both the axial and sagittal views. This structural information was gathered as part of a broader study where volumetric structural, resting‐state functional and diffusion weighted MRI scans were also performed to study brain development within the developing Human Connectome Project 26. Written informed consent for each participant was obtained from someone with parental responsibility prior to them being scanned. Main sequence parameters and number of completed acquisitions for each modality and orientation are reported in Table 1.

**Table 1:** Parameters, Timings, and Number of Studied Cases for Each of the Multi‐Slice Sequences.

Modality	*T*_2_ fast spin echo		*T*_1_ inversion recovery fast spin echo	
View	Axial	Sagittal	Axial	Sagittal
# cases	513	513	430	427
Field of view ( ${\text{mm}}^{3})$	145×119.2×100	145×145×107.2	145×122.4×100	145×145×107.2
Read/phase/slice	AP/RL/SI	SI/AP/RL	AP/RL/SI	SI/AP/RL
SENSE factor ( $N_{2}/K_{2}$)	2.04	2.49	2.16	2.56
# slices (*P*)	125	134	125	134
Acquisition matrix ( $K_{1}\times K_{2}$)	180 × 72		180 × 70	
Fast spin echo factor (*E*)	12		7	
# shots (*S*)	6		10	
Scan duration ( s)	192		345.24	
$T_{R}$/ $T_{E}$/ $T_{I}$ ( ms)	12,000/156/—		4795/8.7/1740	
Resolution ( ${\text{mm}}^{3}$)	0.81×0.81×1.60 (slice overlap 0.80)			

**Figure 2:**
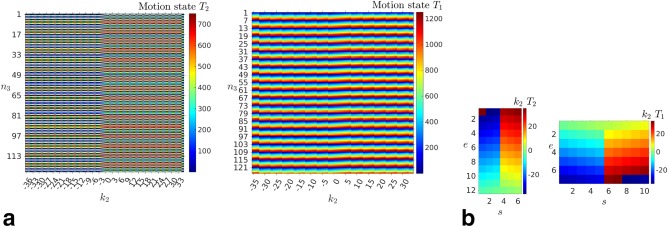
Illustration of the slice and shot sampling structure for both the *T*_2_ and *T*_1_ sequences. **a**: Acquisition order in time. Colorbar shows the order in which the scanned information is acquired, so the images reflect the order in which the phase encode's (horizontal axis) and slices (vertical axis) are acquired during scan time. This acquisition structure is used in our method to define a set of motion states for which corresponding acquired information is assumed to be subject to negligible motion inconsistencies. **b**: Sampling structure in the phase encode direction. Colorbar shows the phase encode ordering, so the images reflect the order in which the different shots (horizontal axis) and echoes (vertical axis) covered the acquired k‐space. This spectral acquisition structure is used by our method to infer motion estimates from the partial k‐space information corresponding to each shot.

The shot and slice sampling structure of our sequences is sketched in Figure 2 for the axially acquired data (similar patterns are observed for the sagittal data, with differences only in the number of slices). Figure 2a shows the different interleaving patterns used for *T*_2_ and *T*_1_, where a hierarchical interleave is used both in the phase encode and slice directions. The differences in the interleaving strategies for the *T*_2_ and *T*_1_ cases can be explained by their different targeted contrasts which are achieved by manipulating their *T*_R_ and *T*_E_. To help interpretation, the echo and shot structure for k‐space sampling are included in Figure 2b. In the *T*_2_ case, the center of the spectrum is reached at the end of the echo train, whereas in the *T*_1_ case it is acquired at the beginning.

### Algorithmic Implementation

Certain term rearrangements, simplifications, and acceleration strategies are necessary to alleviate the computational complexity of the formulation proposed in Equation (2):

1. For common Cartesian acquisitions, such as the fast spin echo sequences described before, the spectral measurements are arranged along lines, so the DFT along the readout direction can be separately precomputed. Assuming that the readout corresponds to the first dimension, *i* = 1, we have:
  (9) $$(\hat{x},\hat{T})=\underset{x,T}{\text{argmin}}||A^{\left(s\right)}F^{\left(2,3\right)}A^{\left(r\right)}HSTx-F^{(1)H}y|{|}_{2}^{2}+\lambda ||Wx|{|}_{2}^{2},$$
  where $F^{\left(2,3\right)}$ is comprised of submatrices of size $K_{2}K_{3}\times N_{2}Q$ corresponding to the 2D DFT with applied k‐space resampling and multiband blipping pattern.
2. The datasets used to validate our method have been acquired using only in‐plane acceleration, without simultaneous multi‐slice excitations. In this case, the multiband factor is *Q* = 1, the number of excitations to cover the FOV corresponds to the number of excited slices, *R* = *P*, and the blipping pattern cycling period is also $K_{3}=1$. Thus, we can write
  (10) $$(\hat{x},\hat{T})=\underset{x,T}{\text{argmin}}||A^{\left(s\right)}F^{\left(2\right)}A^{\left(r\right)}HSTx-F^{(1)H}y|{|}_{2}^{2}+\lambda ||Wx|{|}_{2}^{2},$$
  where $F^{\left(2\right)}$ is comprised of submatrices of size $K_{2}\times N_{2}$ corresponding to the 1D DFT with applied phase encode resampling.
3. A common setting in MRI reconstruction is that the number of reconstructed slices is matched with the number of acquired slices, so $R=P=N_{3}$. For high resolution applications, another reasonable assumption is that the coil sensitivities remain approximately constant along the slice support, as they usually present a slow spatial variation. In consequence, they can commute with the slice profile and excitation matrices and be applied on a slice by slice basis:
  (11) $$(\hat{x},\hat{T})=\underset{x,T}{\text{argmin}}||A^{\left(s\right)}F^{\left(2\right)}SA^{\left(r\right)}HTx-F^{(1)H}y|{|}_{2}^{2}+\lambda ||Wx|{|}_{2}^{2},$$
  where now **H** is comprised of $N_{1}N_{2}SR\times N_{1}N_{2}SR$ submatrices of size $N_{3}\times N_{3},\;A^{\left(r\right)}$ is comprised of $N_{1}N_{2}SR\times N_{1}N_{2}SR$ submatrices of size $1\times N_{3}$ that take the value 1 if *r* = *n*_3_ and are 0 otherwise.
4. As the slice profile filter **H** can be assumed to have approximately compact spatial support, the rigid transformation **T** can be applied to a slab in the surroundings of the excited slice that it affects. The number of slices in this slab, which we define to be 2V+1, should be given by the maximum amount of motion to be expected in the through‐plane direction plus the spatial support of the slice profile. We can express the slab extraction operation by a matrix **U** and write:
  (12) $$(\hat{x},\hat{T})=\underset{x,T}{\text{argmin}}||A^{\left(s\right)}F^{\left(2\right)}SA_{U}^{\left(r\right)}H_{U}T_{U}Ux-F^{(1)H}y|{|}_{2}^{2}+\lambda ||Wx|{|}_{2}^{2},$$
  where **U** is comprised of $N_{1}N_{2}\times N_{1}N_{2}$ submatrices of size $(2V+1)R\times N_{3}$ that take the value 1 if $|n_{3}-r|\leq V$ and 0 otherwise, $T_{U}$ is comprised of *SR* × *R* unitary submatrices of size $N_{1}N_{2}(2V+1)\times N_{1}N_{2}(2V+1),\;H_{U}$ is comprised of submatrices of size (2V+1)×(2V+1), and $A_{U}^{\left(r\right)}$ is comprised of submatrices of size 1×(2V+1) that take the value 1 if v=V+1 (i.e., at the center of the slab) and 0 otherwise. We have set *V* = 3 in our tests.
5. Coil information is compressed 27 to 95% of its energy to accelerate computation, which has been observed to have a minor impact in signal to noise ratio and ability to resolve motion. Thus, if we respectively denote by $\bar{S}$ and $\bar{y}$ the compressed coil sensitivities and measurements, we have:
  (13) $$(\hat{x},\hat{T})=\underset{x,T}{\text{argmin}}||A^{\left(s\right)}F^{\left(2\right)}\bar{S}A_{U}^{\left(r\right)}H_{U}T_{U}Ux-F^{(1)H}\bar{y}|{|}_{2}^{2}+\lambda ||Wx|{|}_{2}^{2},$$
6. A multi‐resolution strategy is adopted that progressively incorporates high‐frequency components into the formulation. Two resolution levels are used with an in‐plane subsampling ratio of 2. The alternation between motion estimation and reconstruction is not applied at the finest scale to accelerate computations, which is observed to have a minor impact in the quality of obtained reconstructions.
7. A graphical processing unit (GPU) version of the algorithm is used in practice.

Coil sensitivity maps are estimated from a separate reference scan 28. A spatial mask is obtained from the reference scan and used to constrain the reconstruction to be zero in the background. In the presence of strong motion, planning and masking have to be made conservative enough so as to prevent the imaged structures from moving outside the prescribed region of interest. Finally, outlier rejection threshold (*τ*) and weight of reconstruction stabilizer (*λ*) parameters have been tuned by visual inspection of exemplary volumes from within the pilot data acquired in our study and kept constant for all subjects. The former has been set to τ=1.2 for all the modalities and orientations explored. The latter has been selected taking account of signal to noise ratio/resolution trade‐off for each modality but kept the same for different orientations. Using this scheme, motion corrected reconstructions are obtained with a GEFORCE GTX TITAN X GPU in 30 min to 3 h per volume, with computation times mainly dependent on the level of motion.

### Image Quality Assessment

Two metrics previously suggested as good indicators of image quality degradation in the presence of motion 15, 29, 30 have been used to quantitatively assess the relative image quality of uncorrected and corrected reconstructions in our cohort:

- The $\ell_{1}$‐norm of a wavelet decomposition of the images 29, $V_{a}=||W_{\text{Db}-a}^{3}x|{|}_{1}$, with $W_{\text{Db}-a}^{3}$ denoting the *a*‐vanishing moments Daubechies (Db) wavelet decomposition at level 3 and a={1,…,4}.
- The gradient entropy (GE), $V_{5}=H(|\nabla x|)$, of the images, which was shown to have the strongest correlation with observer quality scores in Ref. 30.

As we are interested in the motion correction ability, to isolate the effects coming from the thick slice profiles, we have compared the results of applying a conjugate gradient‐SENSE reconstruction that incorporates our proposed deconvolution of the slice profiles without any motion or outlier modelling, non motion corrected with slice profiles (NMC‐SP), and the fully motion corrected (MC) results. A paired right‐tailed sign test against the null hypothesis that the median of the difference of these metrics $V_{a},a=\{1,\ldots ,5\}$, with and without motion correction is lower than zero or zero is performed to assess whether the motion corrected reconstruction effectively improves the sparsity of the wavelet coefficients and decreases the entropy of the reconstructed image gradient. In addition, we report the distribution of the relative change of the different metrics from corrected to uncorrected reconstructions, given by $\sigma_{a}=\frac{2(V_{a}^{\text{MC}}-V_{a}^{\text{NMC}-\text{SP}})}{V_{a}^{\text{MC}}+V_{a}^{\text{NMC}-\text{SP}}}$, as well as the improvement ratio *r*, the fraction of cases in which the corresponding metric decreased after correction, which would suggest a data quality improvement.

## RESULTS

### Visual Validation

**Figure 3:**
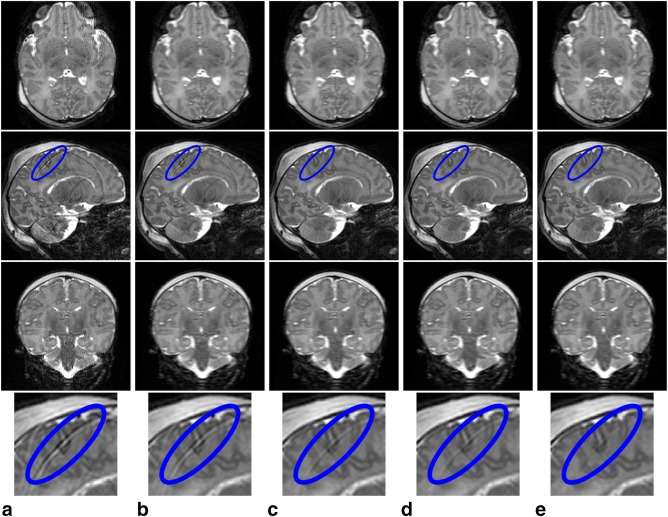
Comparison of results produced in a mildly artifacted *T*_2_ axial acquisition when different components of the proposed motion corrected reconstruction method are omitted. **a**: Conventional uncorrected SENSE reconstructions. **b**: Uncorrected reconstructions when integrating the slice profile filter. **c, d**: Motion corrected reconstruction excluding one element at a time: (c) no within‐plane motion model; (d) no through‐plane motion model. **e**: Full motion corrected reconstructions. From top to bottom, axial view, sagittal view, coronal view, and magnified results within the area enclosed in blue in the sagittal view.

**Figure 4:**
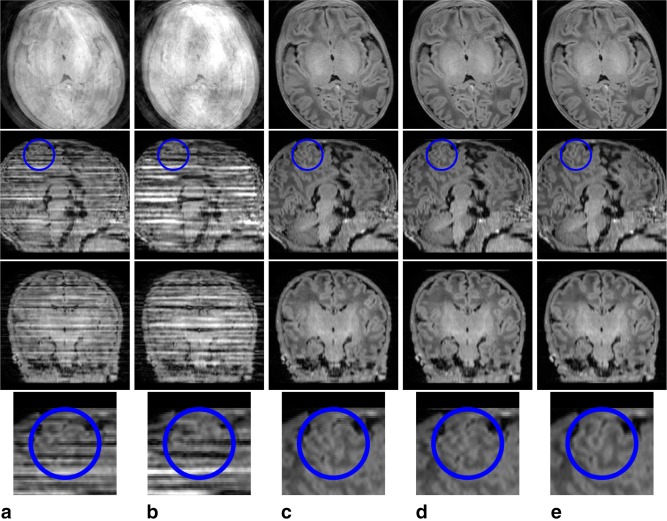
Comparison of results produced in a highly artifacted *T*_1_ axial acquisition when different components of the proposed motion corrected reconstruction method are omitted. **a**: Uncorrected reconstructions when integrating the slice profile filter. **b–d**: Motion corrected reconstruction excluding one element at a time: (b) no outlier rejection strategy; (c) no within‐plane motion model; (d) no through‐plane motion model. **e**: Full motion corrected reconstructions. From top to bottom, axial view, sagittal view, coronal view, and magnified results within the area enclosed in blue in the sagittal view.

To show the benefits that follow from including the main elements of the proposed framework, two levels of motion corruption and two different native acquisition planes are illustrated in Figures 3 and 4, which show respectively a mildly damaged *T*_2_‐weighted sagittal acquisition and a grossly damaged *T*_1_‐weighted axial acquisition. In each case, the separate rows depict transverse, sagittal, and coronal sections through the stacks of slices, as well as magnified areas to highlight fine detailed image differences, and the columns present different configurations of the motion corrected reconstruction with or without its main constituents: slice profile model, within‐plane motion, through‐plane motion and outlier rejection.

In the mildly corrupted *T*_2_ sagittal acquisition of Figure 3, the introduction of the slice profile in the reconstruction model (Fig. 3b) tends to suppress the saw‐tooth boundary artifacts observed in non‐native views (i.e., axial and coronal views) for standard SENSE reconstruction (Fig. 3a). However, localized artifacts are still observed in the sagittal view. The strength of these artifacts gets reduced when either through‐plane (Fig. 3c) or within‐plane (Fig. 3d) motion correction is introduced, as illustrated by the blue ellipse area. Finally, we observe how when both components of motion are incorporated into the model, the artifacts get further reduced and the through‐plane consistency of the data gets improved (Fig. 3e). In this example, we have not included the results without outlier rejection as the inclusion of this feature did not influence the results.

In the highly corrupted *T*_1_ axial acquisition of Figure 4, the reconstruction alternatives that do not include outlier rejection (Fig. 4a,b) are unable to recover consistent information, as the magnetization properties of certain shots can be severely altered due to motion. Without outlier rejection, application of motion correction may distribute magnetization artifacts in the slice direction, so the quality of motion corrected data (Fig. 4b) is not necessarily superior to that of non‐corrected data (Fig. 4a). Although not included here, a similar effect is observed when comparing standard SENSE reconstructions and reconstructions using the slice profile model, where corrupted slices will damage neighboring slices. When outlier rejection is introduced (Fig. 4c,d,e), shots with irreconcilable data are effectively discarded and the reconstructed information appears more consistent. However, interslice misalignments are observed in the blue circle area when either within‐plane (Fig. 4c) or through‐plane (Fig. 4d) motion is not considered in the motion model, whereas the cortical boundaries appear better aligned when both components of motion are incorporated (Fig. 4e).

**Figure 5:**
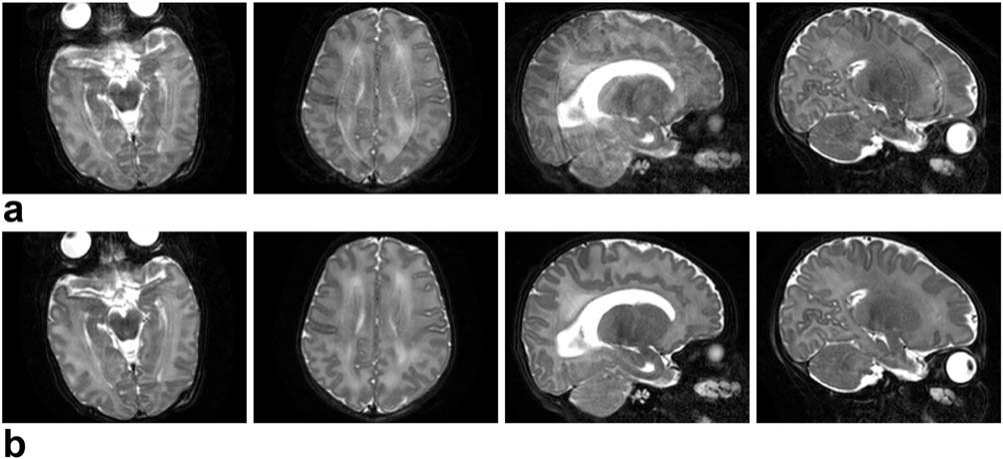
Snapshots of *T*_2_ reconstructions for different subjects, orientations, and locations in the brain. Each column corresponds to a different subject example. **a**: Uncorrected reconstructions. **b**: Corrected reconstructions.

**Figure 6:**
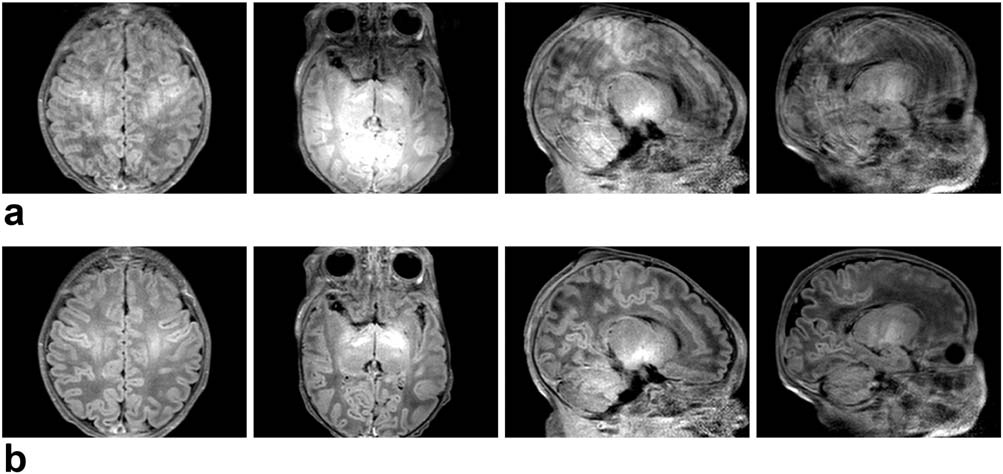
Snapshots of *T*_1_ reconstructions for different subjects, orientations, and locations in the brain. Each column corresponds to a different subject example. **a**: Uncorrected reconstructions. **b**: Corrected reconstructions.

Further examples of the ability to correct a variety of artifacts in the acquired slices are shown in Figure 5 (*T*_2_ case) and Figure 6 (*T*_1_ case), where uncorrected versus corrected reconstructions are shown in the native slice orientations. Different subjects and slice locations in the brain are illustrated in these Figures, showing the flexibility of the technique (see Supporting Information and refer to the description in “Quantitative Validation” section for further examples of the performance of the method blindly extracted from the database).

### Quantitative Validation

**Figure 7:**
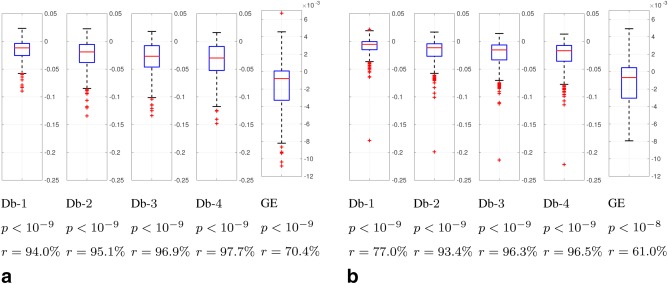
Box plots of relative metric change *σ*, *P*‐values of a paired right‐tailed sign test, and percentage of cases *r* in which the metric decreased for the $\ell_{1}$ norm of Db wavelet decompositions and for the GE in motion corrected versus uncorrected reconstructions. Negative values in the paired box plots indicate a decrease in the corresponding metrics when applying motion correction, which has been documented as associated with an improvement in image quality (29,30). **a**: Axial *T*_2_. **b**: Sagittal *T*_2_.

**Figure 8:**
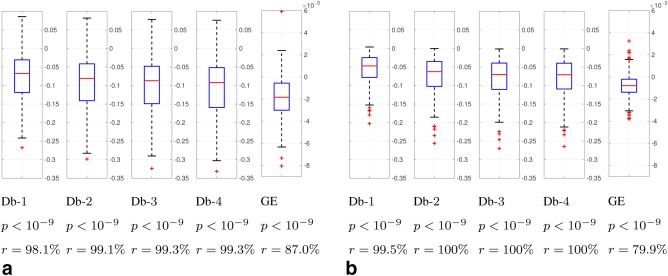
Box plots of relative metric change *σ*, *P*‐values of a paired right‐tailed sign test, and percentage of cases *r* in which the metric decreased for the $\ell_{1}$ norm of Db wavelet decompositions and for the GE in motion corrected versus uncorrected reconstructions. Negative values in the paired box plots indicate a decrease in the corresponding metrics when applying motion correction, which has been documented as associated with an improvement in image quality (29,30). **a**: Axial *T*_1_. **b**: Sagittal *T*_1_.

The data quality of motion corrected and uncorrected reconstructions using the metrics described in “Image Quality Assessment” section is compared in Figures 7 (*T*_2_ case) and 8 (*T*_1_ case). Box plots of the distribution of the relative metric change from corrected to uncorrected reconstructions *σ*, significance levels *P*, and improvement ratios *r* for each metric are included. Results for the whole dataset of 1883 brain volumes from 517 examinations are shown for different views and contrasts, axial *T*_2_ (Fig. 7a), sagittal *T*_2_ (Fig. 7b), axial *T*_1_ (Fig. 8a), and sagittal *T*_1_ (Fig. 8b). They correspond to different shot and slice sampling structures as shown in Figure 2. *T*_2_ sequences are acquired at the beginning of the study whereas *T*_1_ sequences are acquired at the end. Total scan duration is also different for *T*_2_ and *T*_1_, as shown in Table 1. Moreover, different relative strengths of within‐plane and through‐plane motion may be expected for different orientations. In addition, a representative sample of reconstructions for different contrasts has been included as Supporting Information and made available in the online version of the article. These include 10 volumes for each modality sampled at percentiles {0.05,0.15,0.25,0.35,0.45,0.55,0.65,0.75,0.85,0.95} of $-(\sigma_{4}^{\text{axial}}+\sigma_{4}^{\text{sagittal}})$, selected as an indicator of the degree of motion correction for a given modality. On the one hand, this material is intended to show the agreement between the image quality metric *σ*_4_ and actual motion correction, with lower percentiles correlated with small level of correction (either due to inability to correct or due to small corruption). On the other hand, it is conceived to show unbiased information about the overall performance of the method in our database; aside from blindly choosing cases using the percentile rule, shown slices have also been picked out blindly at FOV/2 in the axial and coronal views and at FOV/3 in the sagittal view. Results for *T*_2_ and *T*_1_ modalities are respectively included in Supporting Figures S1–S10 and S11–S20.

Differences between corrected and uncorrected reconstructions in Figures 7 and 8 are highly significant ( P≪0.05) for all metrics, contrasts, and orientations. Thus, they show the ability of the motion correction method to effectively improve the compressibility and minimize the entropy of the gradient of reconstructed images, which, we interpret, is derived from its ability to reduce motion artifacts (see Supporting Information for visual verification). Larger improvement ratios (*r*) are generally obtained for *T*_1_ than for *T*_2_ scans. Larger improvement ratios can be due to larger overall motion artifacts in the sample or better performance of the method. We think that, in this case, we are in the first situation, as uncorrected *T*_1_‐weighted images usually show more prominent motion artifacts than *T*_2_‐weighted images (see Supporting Information). This may be due to the former being acquired at the end of the one hour examination protocol, with attendant risk that the unsedated neonates may stir, their longer scan duration as compared to the *T*_2_ scans, and/or the specific inner time structure of this sequence, which involves the recovery from inversion. Improvement ratios are substantial for the wavelet measure and more moderate for the GE. We think this may be related with the wavelet metric showing a reduction when ghosts are corrected, which we have observed to be usual in our database, while the GE metric may be mainly an indicator of blurring, a less common artifact in the tested sequences.

### Assembling Different Orientations

**Figure 9:**
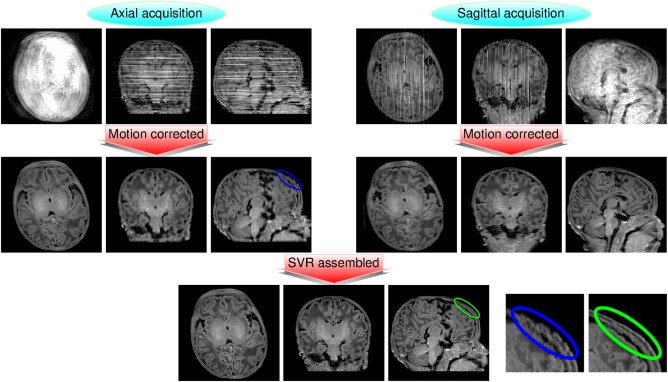
Slice to volume reconstruction‐based assembling of motion corrected information of different views for suppression of residual artifacts and isotropic resolution: *T*_1_ example. Volumetric data consistency is substantially improved for each of the views after applying our method, but residual motion may still be present due to remaining inconsistencies between slices, and non‐native views may appear blurred as compared to native views. After slice to volume reconstruction correction, information is made consistent between views and a nearly isotropic representation of the imaged volume is obtained. In the bottom right corner, we show a magnified example comparing the results for the skull in the sagittal slice of the axial acquisition (left image, enclosed in blue) versus the corresponding results after view assembling (right image, enclosed in green), with residual motion inconsistencies strongly suppressed in the latter.

Despite being able to significantly diminish the spurious information introduced by motion inconsistencies, our method operates separately on each view, so the resulting volumetric image resolution may be biased by the acquired orientation, particularly in cases of large through‐plane motion. To get an isotropic representation of the imaged volumes, we have deployed the slice to volume reconstruction method in Ref. 20 to assemble the information coming from different views. This method is able to correct for motion inconsistencies and intensity biases among slices and provides a super‐resolved representation of the information coming from thick slices acquired in different orientations, so it matches very well with the residual corrections that may be required after our method. An example of the proposed pipeline for robust neonatal structural brain imaging is shown in Figure 9. More examples of view assembling are included as Supporting Information. In our project, resulting *T*_2_ volumes are used for brain segmentation and *T*_1_ volumes are later mapped to the *T*_2_ space to study brain maturation.

## DISCUSSION

Proposed method for motion corrected reconstructions in multi‐shot multi‐slice imaging is based on combining a strategy to estimate the rigid motion states of the imaged structure on a per‐shot basis on top of a conjugate gradient‐SENSE reconstruction that accounts for slice profiles to integrate the volumetric information of the acquisition. This article is mainly focused on the ability to estimate the within‐plane and through‐plane components of motion while discarding those shots that have intrinsic magnetization differences to get a consistent representation of the imaged volume. This has been tested for different structural modalities and orientations in a large database of neonatal studies obtained from the subjects in natural sleep, when there is a risk of sporadic movement. We have provided evidence of significant improvement both from visual assessment in selected examples and quantitative measures obtained from all available images. The formulation can be applied when using in‐plane acceleration and simultaneous multi‐slice excitation. However, only the former was tested and it is possible that some aspects of the implementation presented in “Algorithmic Implementation” section may need review for efficient reconstruction in the latter case.

Although we have demonstrated certain capability to correct through‐plane motion, full correction at the prescribed resolution may not be possible because subject movement can result in insufficiently dense samples in some regions of the brain. Two complementary main strategies are envisaged to deal with nonuniform sampling. The first is the one adopted in this article: by acquiring additional sets of orthogonal of slices, inherent anisotropy can be corrected either as a post‐processing step or by direct integration of multi‐view information in the formulation. Optimal within‐view acceleration and number of acquired orientations should be properly balanced for efficiency and robustness. The second would be the adoption of prospective motion correction strategies 31 with additional added value in preventing spin‐history effects. In this case, retrospective motion correction techniques as presented here may still be useful in providing correction of cross‐calibration errors 32 or in treating artifacted shots.

Our method builds on a rigid model assumption that is only approximately valid for the brain. Thus, full correction cannot be expected for non‐rigid sources of motion 33 or when the motion description is not unique due to other moving structures within the FOV 2. Issues due to non‐rigid motion in the brain stem or in the neck can be alleviated by encoding using a foot‐head readout and limiting the motion estimates to the brain regions where the rigid body model fits well. This was actually applied in Ref. 15 but we have not found it to be necessary in the testing database used in this article, as the convergence of the algorithm is governed by the larger contrast of the cortical structures and their proximity to the coils. More important in practice was the application of a fat suppression technique to avoid motion inconsistencies due to water‐fat shift. More complex motion models could be incorporated to our matrix formulation similarly to Ref. 14, however, the ability to effectively constrain and solve the problem will be largely application dependent, particularly when dealing with through‐plane motion.

The hard criterion adopted in this work to differentiate between consistent and inconsistent data has been inspired by the type of motion most usually encountered in our application regime, where neonates usually move in sparse bursts during the acquisition, so that data is clearly degraded during those periods. However, this approach may present problems when generalized to other scenarios such as those with more homogeneous motion degradation during the acquisition (for instance for correcting small motions in high‐resolution imaging). Using a weighted least squares technique along the lines of Ref. 34, may allow more effective data integration, but some work is needed on how to best design data uncertainty estimation and control the potentially unstable convergence of the joint motion estimation and reconstruction.

Other future developments could include more comprehensive treatment of (1) the inhomogeneous signal to noise ratio of parallel imaging as given by the array of coils and noise penalty implications of adopted acceleration methods, (2) the uncertainty in sensitivity map estimates, (3) the presence of other potential artifacts, and (4) the inhomogeneous resolution as given by through‐plane motion and, perhaps, discarded shots. In addition, different priors can be used for motion estimation and reconstruction. Thus, a two‐step approach could be envisaged. First, motion is estimated using a linear reconstruction technique as proposed in this article, where the prior is selected for the sake of stability and computability of the procedure. Second, a nonlinear reconstruction technique is used as a final step, where better approximants of the underlying image 35 could be used to integrate the sampled information in the presence of motion.

## CONCLUSIONS

We have presented a method to obtain reconstructions for multi‐shot multi‐slice brain imaging including correction for 3D rigid body motion. The method is an extension of a previously proposed framework for motion estimation from incomplete parallel spectral information in purely volumetric MRI data 15. In this extension, we have provided a general formulation to jointly tackle the within‐plane and through‐plane motion problems as well as a strategy to iteratively solve for motion and get a motion‐free reconstructed volume. The forward modeling framework adopted naturally incorporates parallel imaging acceleration methods (both in‐plane or simultaneous multi‐slice) and addresses through‐slice movement by considering the slice excitation profiles. In addition, a procedure has been proposed to detect and discard artifacted shots. The method has been tested by reconstructing motion corrected images in a large cohort of neonates. Both visual validation and quantitative assessment have demonstrated significant benefits for different contrasts, different acquisition orientations and different shot and slice acquisition orderings. When combined with appropriate acquisition strategies including overlapped slices and multi‐view information, our method has demonstrated to provide a robust motion‐tolerant pipeline for some of the most prevalent structural brain MRI protocols. Thus, although our test data comes from a neonatal population, this proposal may also improve brain imaging performance in general, particularly for non‐compliant populations.

## Supporting information


**Figs. S1–S10**. Examples of correction for *T*_2_ contrast at different levels of the motion correction measure *σ*_4_ showing uncorrected and corrected reconstructions side by side for axial (left) and sagittal (center) slice orientations, along with the result of fusing the two motion corrected reconstructions (right). Examples are provided at regular percentile intervals within the ranked values of *σ*_4_ for the whole cohort. Rank order was determined from the opposite of the sum of *σ*_4_ values for the two acquisitions on each subject, and each acquisition presented is labelled by its individual *σ*_4_ value. Motion artifacts start to be noticeable above percentile 0.55 and within‐view motion correction provides almost complete recovery in all cases (particularly note percentiles 0.75, 0.85, and 0.95). Remaining corruption is mainly in the form of intensity biases, which are amenable to further correction by assembling information from different views via the slice to volume reconstruction method in Ref. 20.
**Figs. S11–S20**. Examples of correction for *T*_1_ contrast at different levels of the motion correction measure *σ*_4_ showing uncorrected and corrected reconstructions side by side for axial (left) and sagittal (center) slice orientations, along with the result of fusing the two motion corrected reconstructions (right). Examples are provided at regular percentile intervals within the ranked values of *σ*_4_ for the whole cohort. Rank order was determined from the opposite of the sum of *σ*_4_ values for the two acquisitions on each subject, and each acquisition presented is labelled by its individual *σ*_4_ value. Motion artifacts start to be noticeable above percentile 0.15. Within‐view motion correction provides significant recovery in all cases (particularly note axially‐acquired data at percentile 0.85 and sagittally‐acquired data at percentile 0.95). In some cases, only partial improvement is achieved by within‐view correction (particularly for axially‐acquired data at percentile 0.75 and sagittally‐acquired data at percentile 0.45). However, remaining corruption and resolution anisotropy are strongly diminished when combining the information from both orientations using the slice to volume reconstruction method in Ref. 20.

## Footnotes

1 We drop bold notation from these DFT's to avoid confusion with the generic DFT matrices in Equations [1] and [2].
