# Supplementary material for: Three‐dimensional motion corrected sensitivity encoding reconstruction for multi‐shot multi‐slice MRI: Application to neonatal brain imaging
Source: Magn Reson Med. 2017 Jun 19;79(3):1365–76. doi: 10.1002/mrm.26796 (PMC5811842; doi:10.1002/mrm.26796)
Supplement: Supplementary file 1 — Figs. S1–S10. Examples of correction for T 2 contrast at different levels of the motion correction measure σ 4 showing uncorrected and corrected reconstructions side by side for axial (left) and sagittal (center) slice orientations, along with the result of fusing the two motion corrected reconstructions (right). Examples are provided at regular percentile intervals within the ranked values of σ 4 for the whole cohort. Rank order was determined from the opposite of the sum of σ 4 values for the two acquisitions on each subject, and each acquisition presented is labelled by its individual σ 4 value. Motion artifacts start to be noticeable above percentile 0.55 and within‐view motion correction provides almost complete recovery in all cases (particularly note percentiles 0.75, 0.85, and 0.95). Remaining corruption is mainly in the form of intensity biases, which are amenable to further correction by assembling information from different views via the slice to volume reconstruction method in Ref. 20. Figs. S11–S20. Examples of correction for T 1 contrast at different levels of the motion correction measure σ 4 showing uncorrected and corrected reconstructions side by side for axial (left) and sagittal (center) slice orientations, along with the result of fusing the two motion corrected reconstructions (right). Examples are provided at regular percentile intervals within the ranked values of σ 4 for the whole cohort. Rank order was determined from the opposite of the sum of σ 4 values for the two acquisitions on each subject, and each acquisition presented is labelled by its individual σ 4 value. Motion artifacts start to be noticeable above percentile 0.15. Within‐view motion correction provides significant recovery in all cases (particularly note axially‐acquired data at percentile 0.85 and sagittally‐acquired data at percentile 0.95). In some cases, only partial improvement is achieved by within‐view correction (particularly for axially‐acquired data at percentile 0. [file MRM-79-1365-s001.pdf]

Supporting Figures S1-S10. Examples of correction for T_2 contrast at different levels of the motion correction measure σ_4 showing uncorrected and corrected reconstructions side by side for axial (left) and sagittal (center) slice orientations, along with the result of fusing the two motion corrected reconstructions (right). Examples are provided at regular percentile intervals within the ranked values of σ_4 for the whole cohort. Rank order was determined from the opposite of the sum of σ_4 values for the two acquisitions on each subject, and each acquisition presented is labelled by its individual σ_4 value. Motion artifacts start to be noticeable above percentile 0.55 and within-view motion correction provides almost complete recovery in all cases (particularly note percentiles 0.75, 0.85 and 0.95). Remaining corruption is mainly in the form of intensity biases, which are amenable to further correction by assembling information from different views via the SVR method in (20).

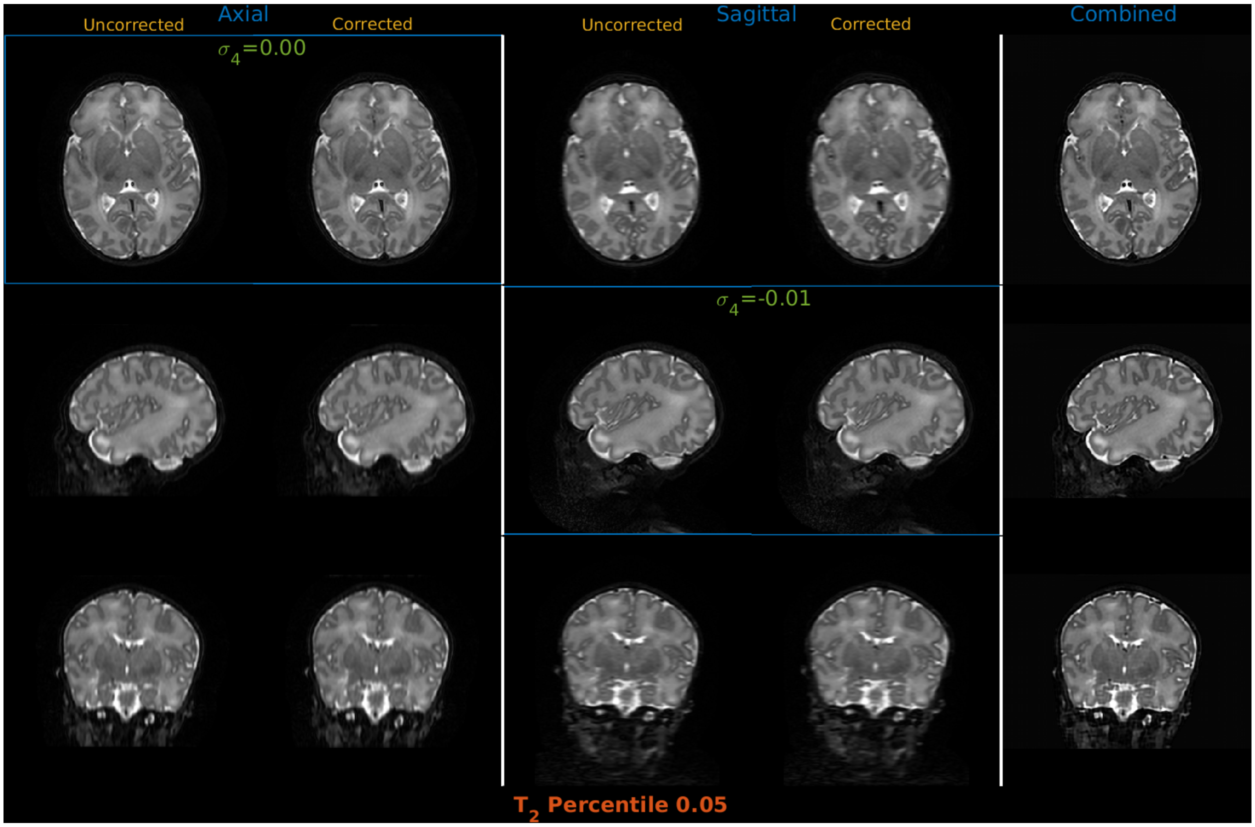

Supporting Figure S1

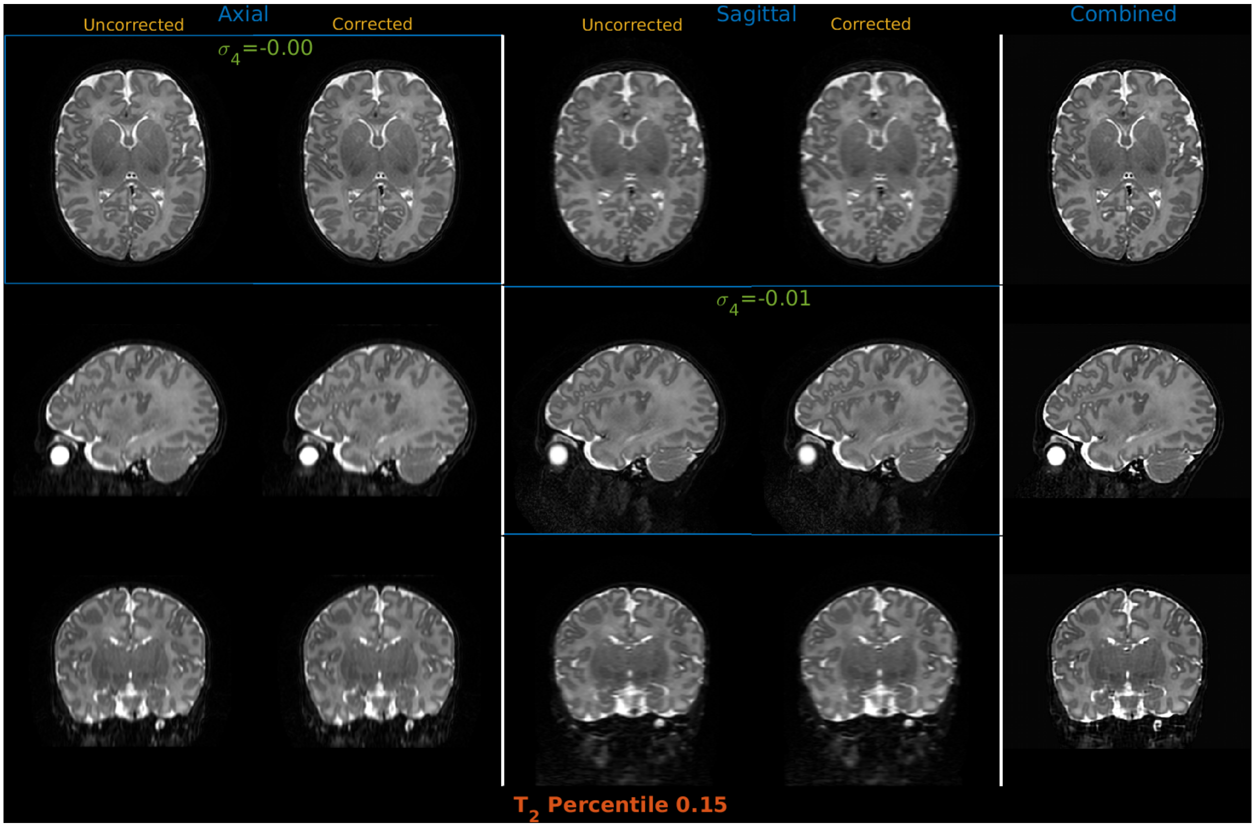

Supporting Figure S2

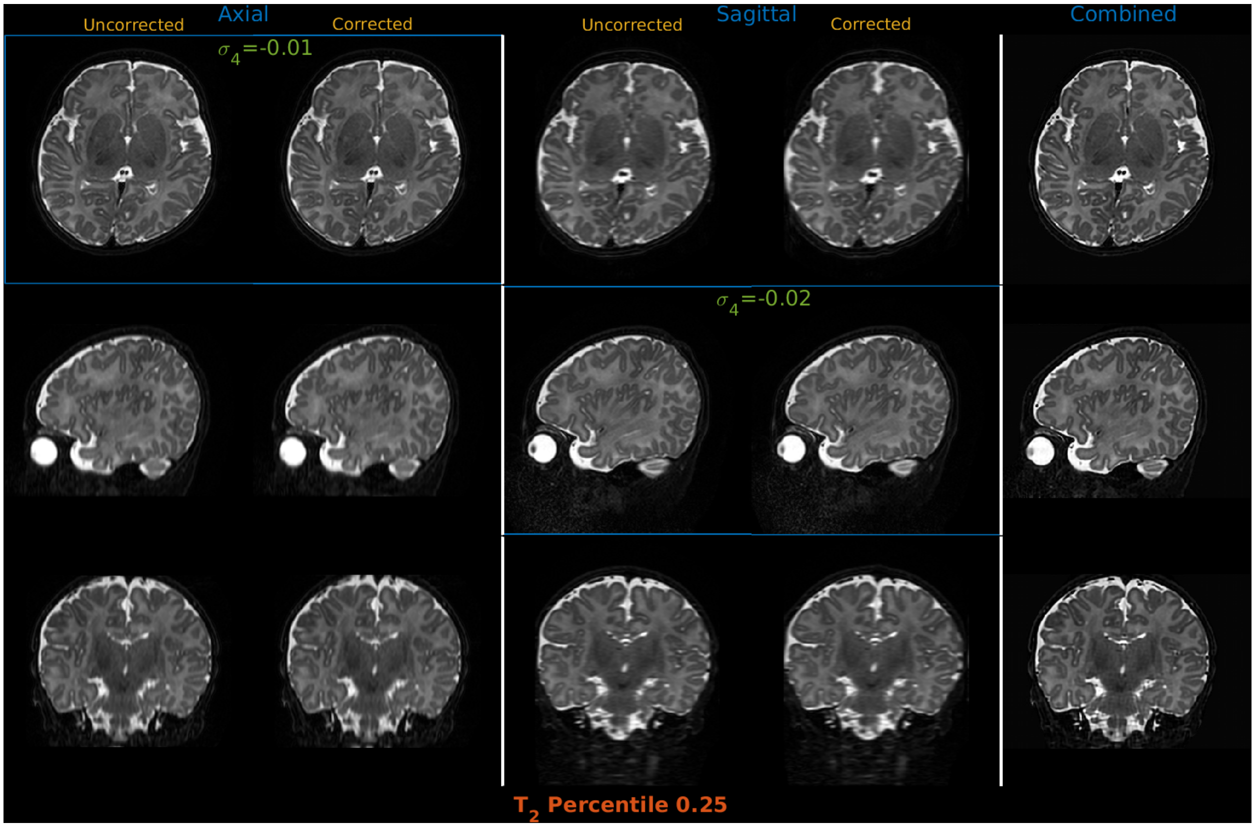

Supporting Figure S3

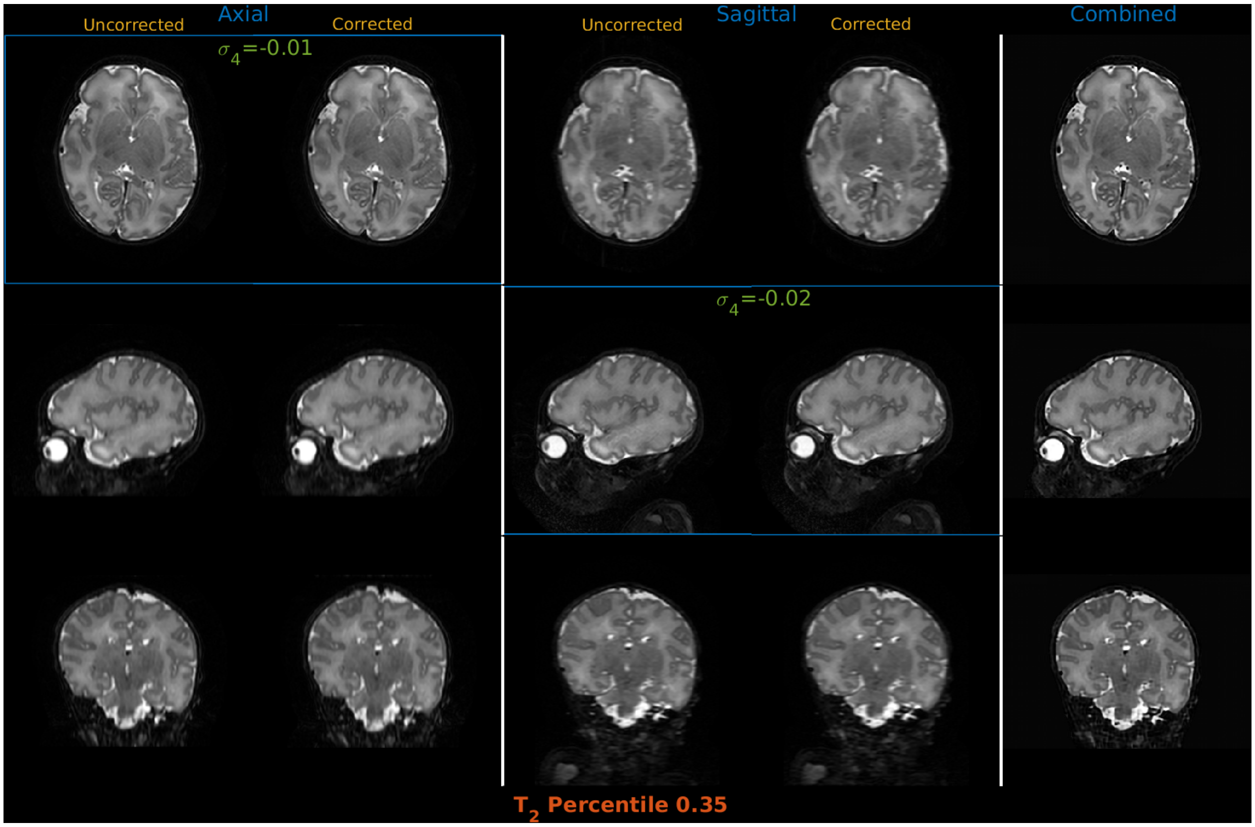

Supporting Figure S4

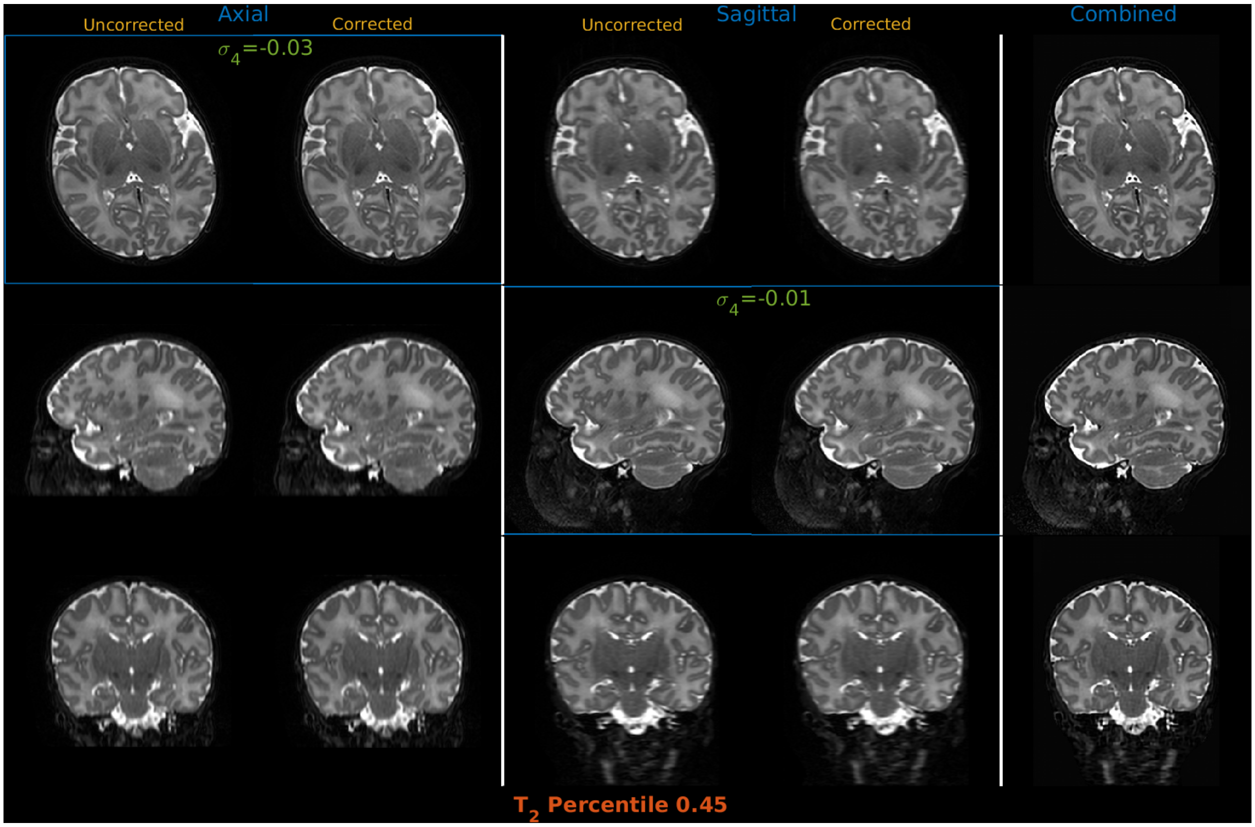

Supporting Figure S5

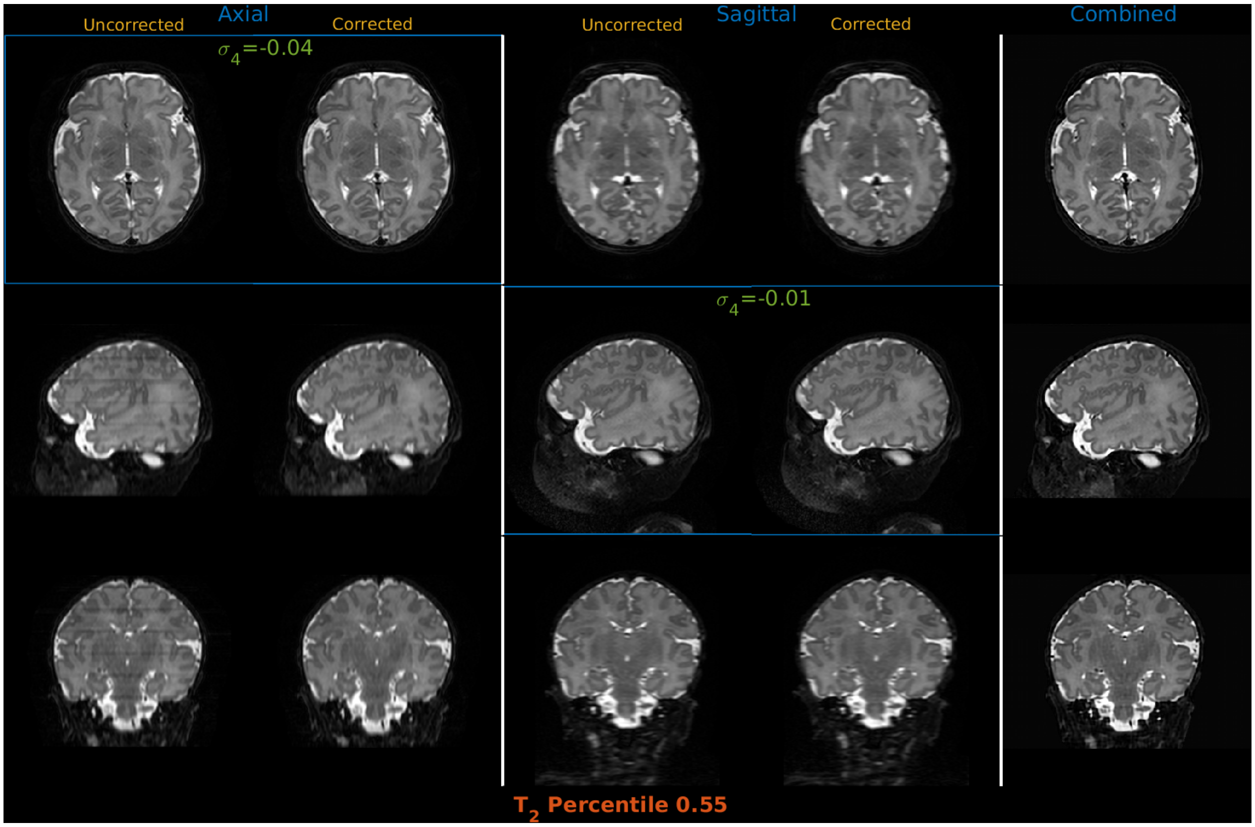

Supporting Figure S6

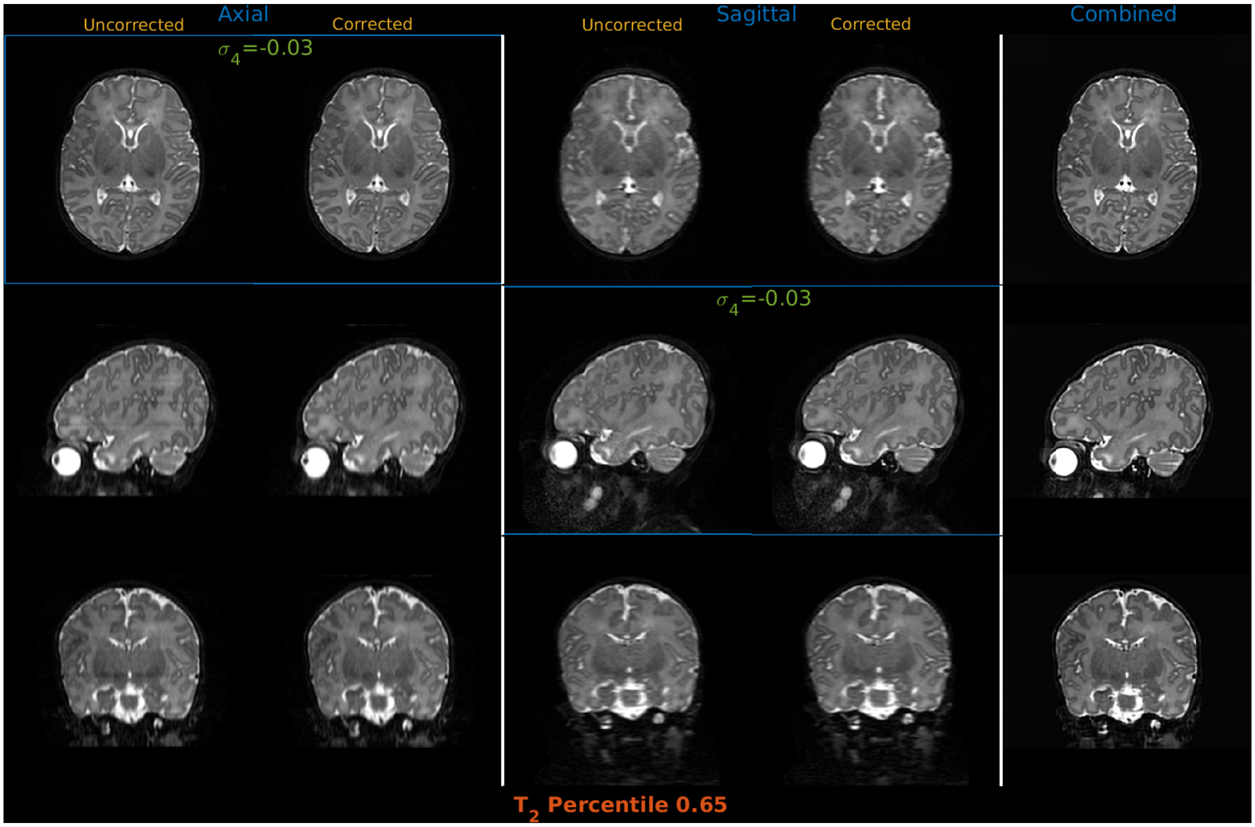

Supporting Figure S7

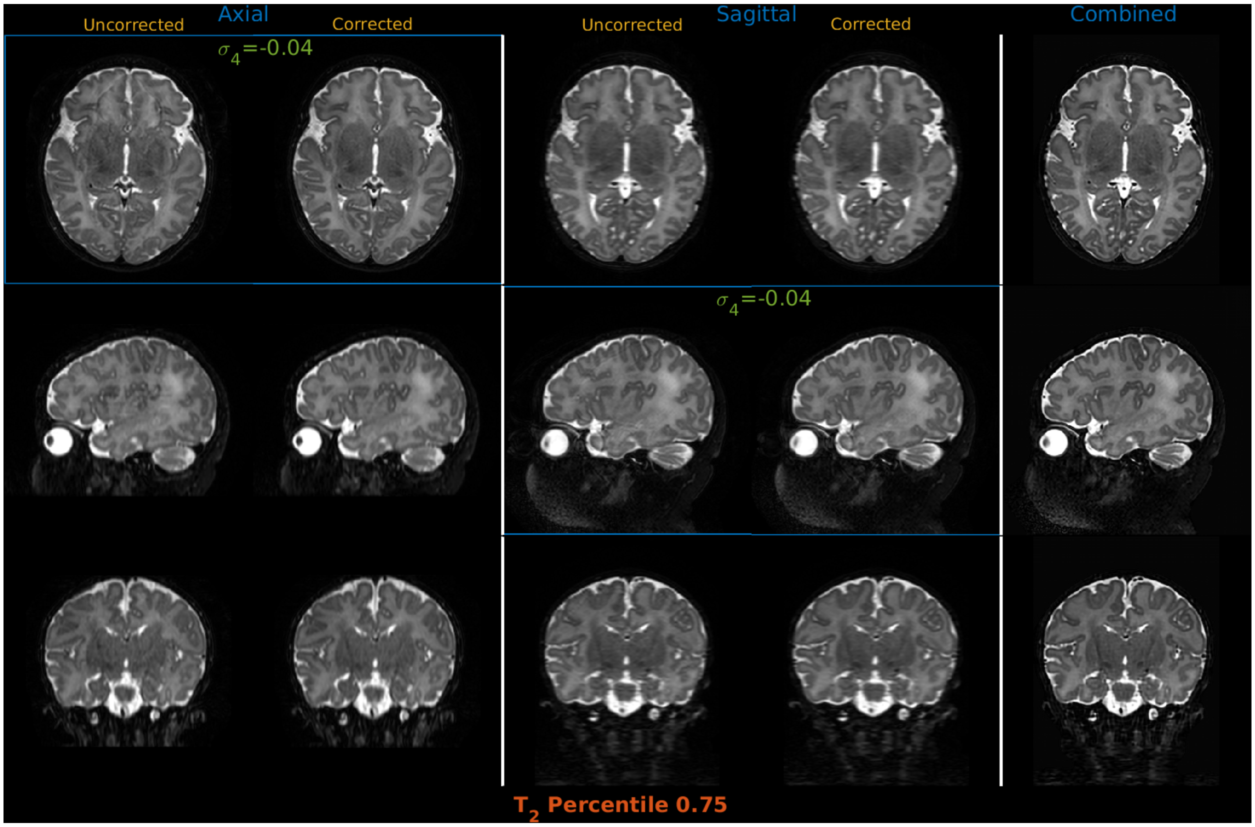

Supporting Figure S8

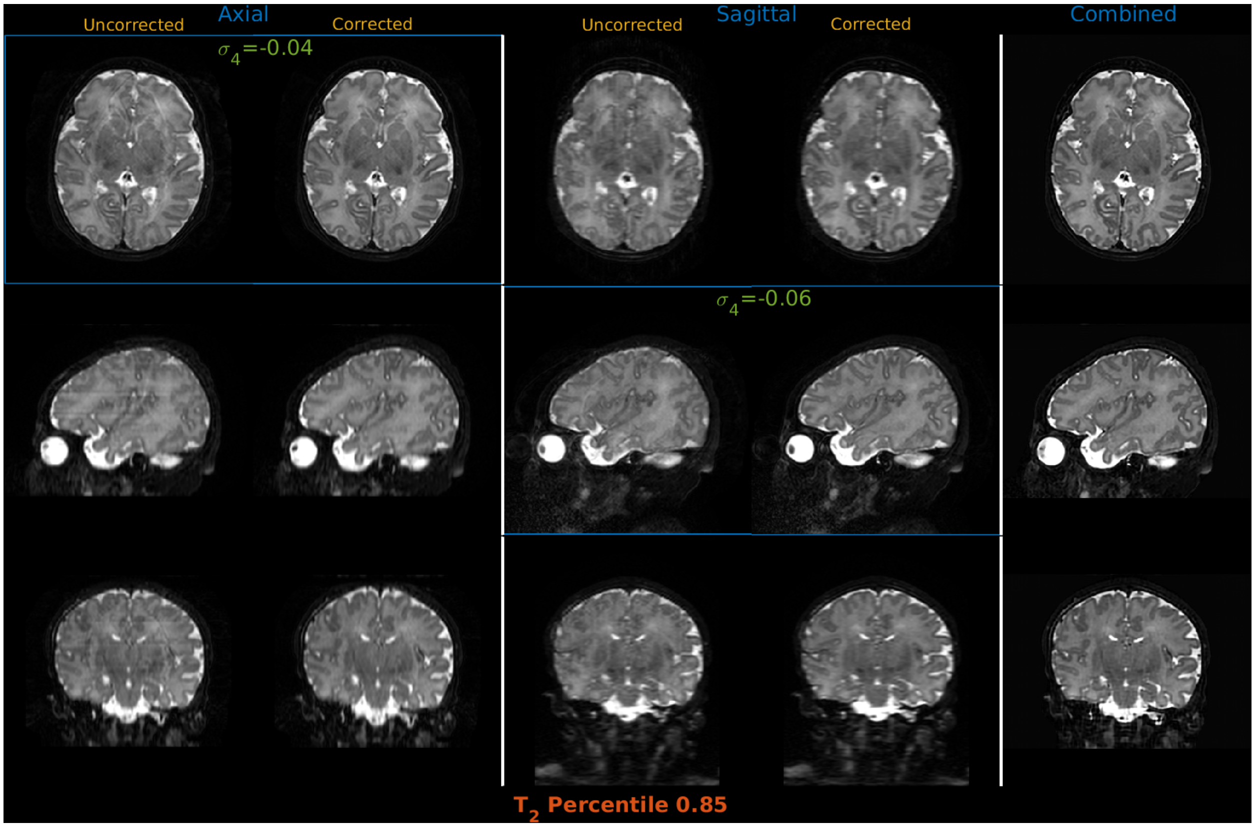

Supporting Figure S9

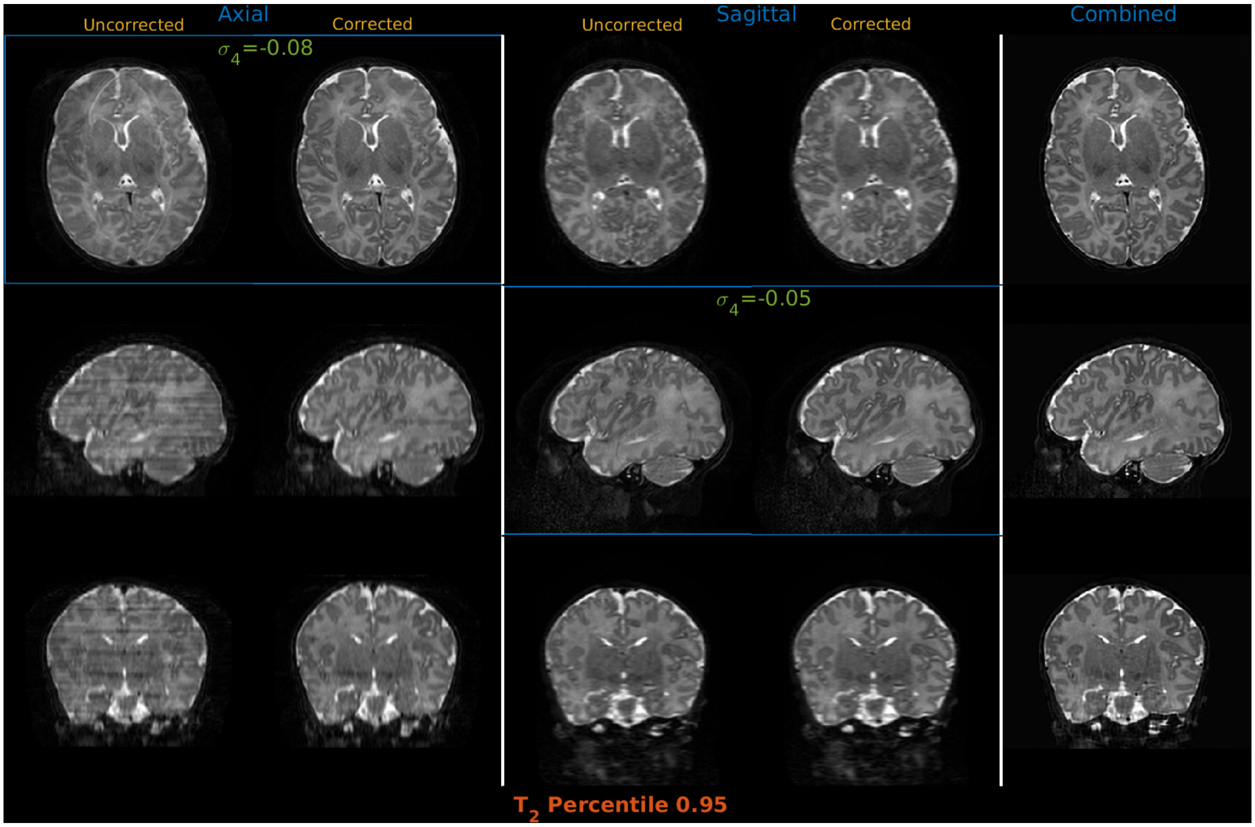

Supporting Figure S10

Supporting Figures S11-S20. Examples of correction for T_1 contrast at different levels of the motion correction measure σ_4 showing uncorrected and corrected reconstructions side by side for axial (left) and sagittal (center) slice orientations, along with the result of fusing the two motion corrected reconstructions (right). Examples are provided at regular percentile intervals within the ranked values of σ_4 for the whole cohort. Rank order was determined from the opposite of the sum of σ_4 values for the two acquisitions on each subject, and each acquisition presented is labelled by its individual σ_4 value. Motion artifacts start to be noticeable above percentile 0.15. Within-view motion correction provides significant recovery in all cases (particularly note axially-acquired data at percentile 0.85 and sagittally-acquired data at percentile 0.95). In some cases only partial improvement is achieved by within-view correction (particularly for axially-acquired data at percentile 0.75 and sagittally-acquired data at percentile 0.45). However, remaining corruption and resolution anisotropy are strongly diminished when combining the information from both orientations using the SVR method in (20).

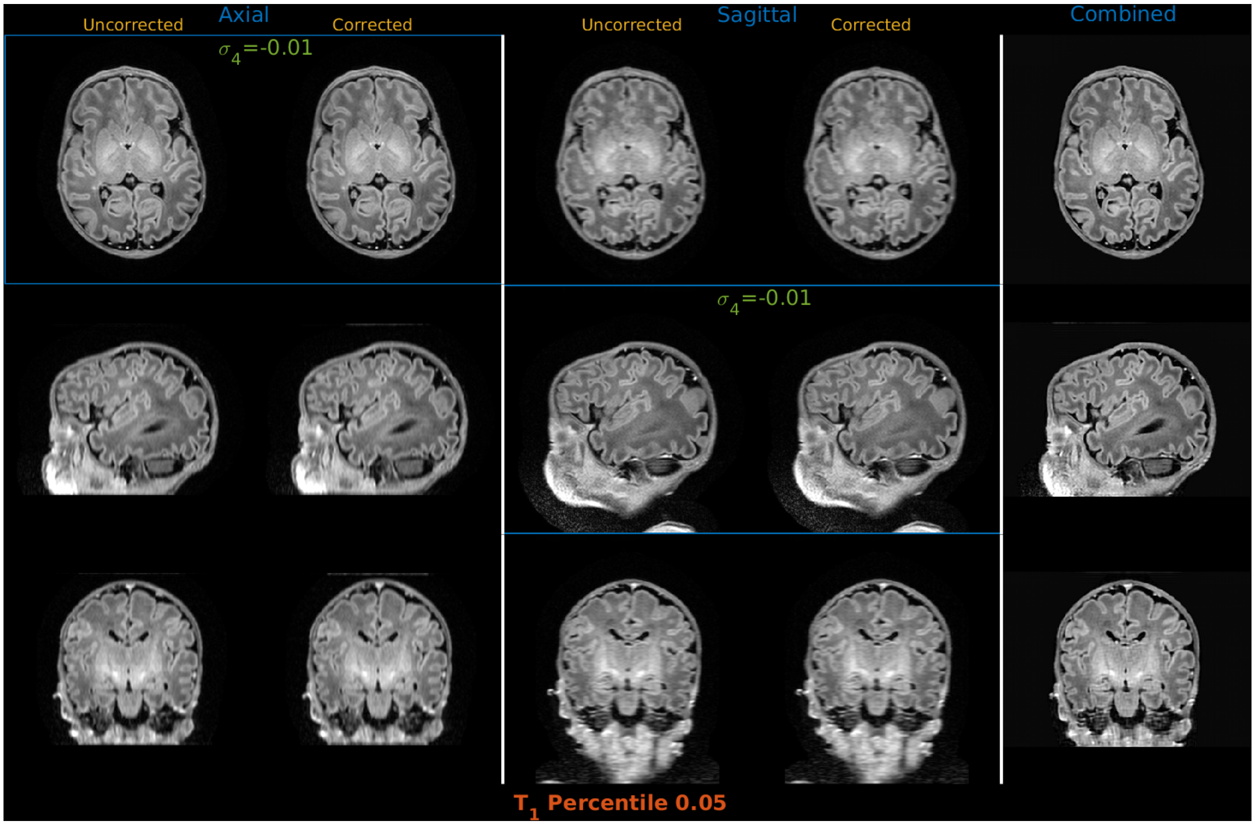

Supporting Figure S11

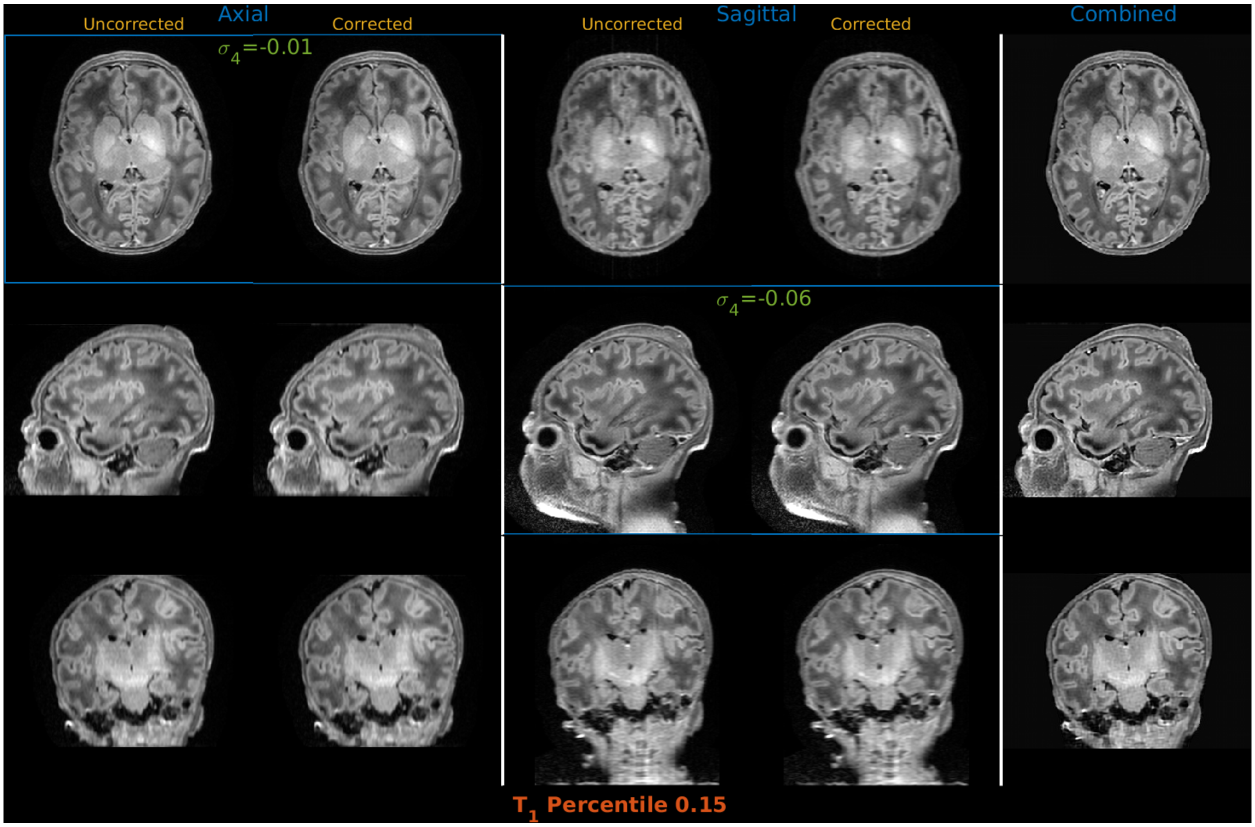

Supporting Figure S12

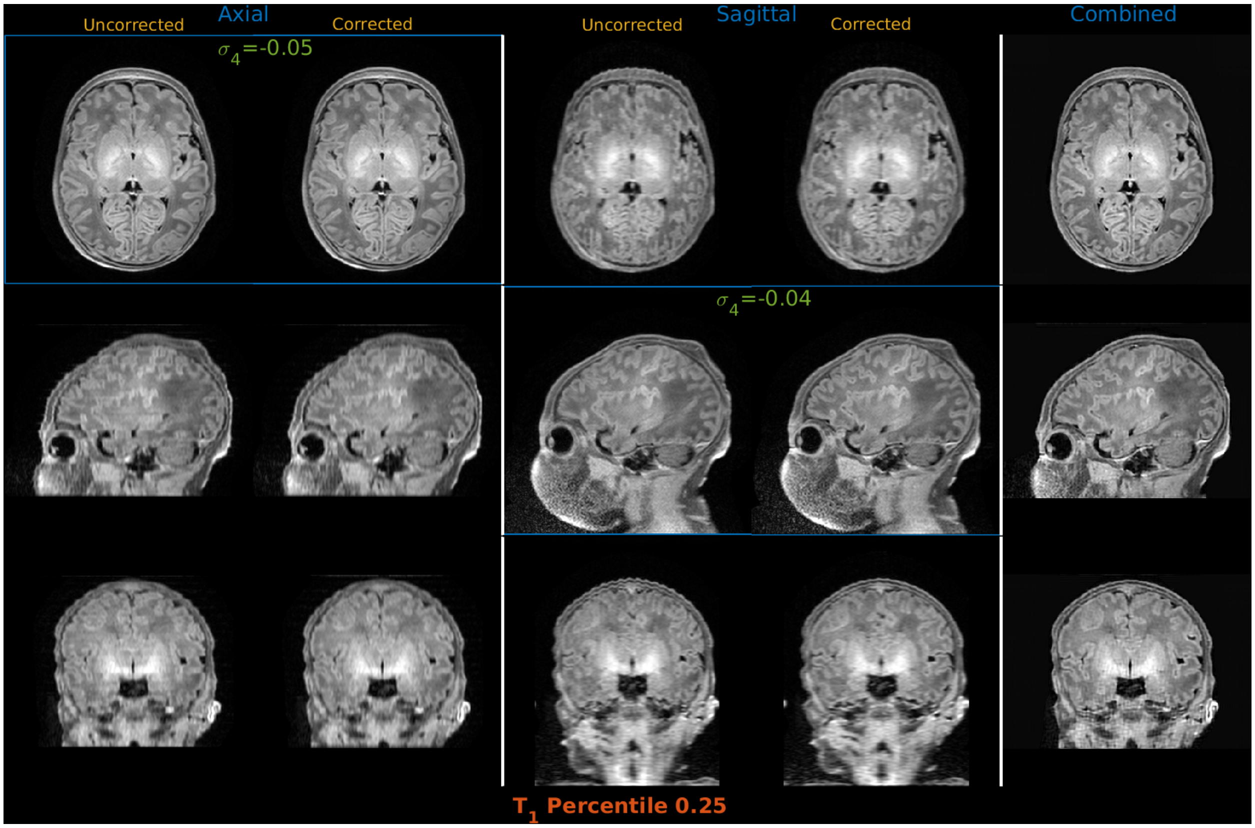

Supporting Figure S13

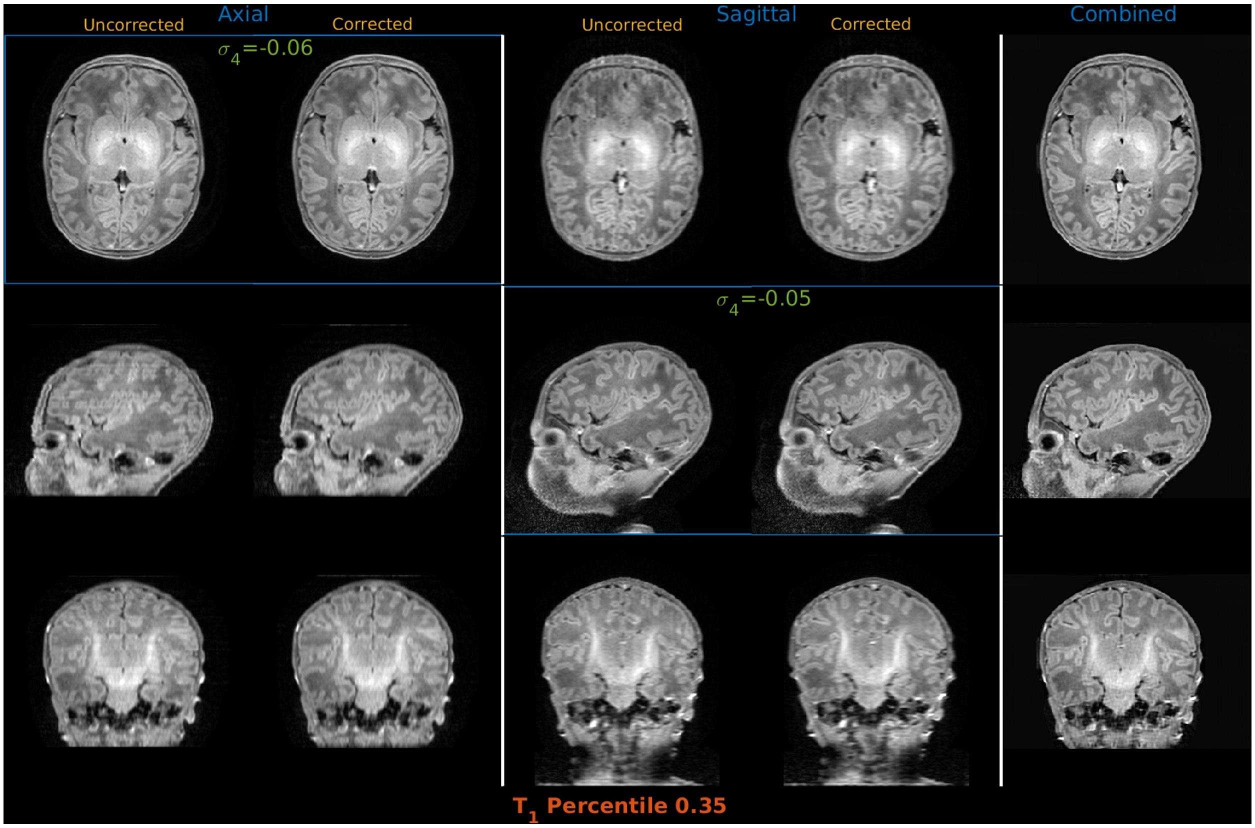

Supporting Figure S14

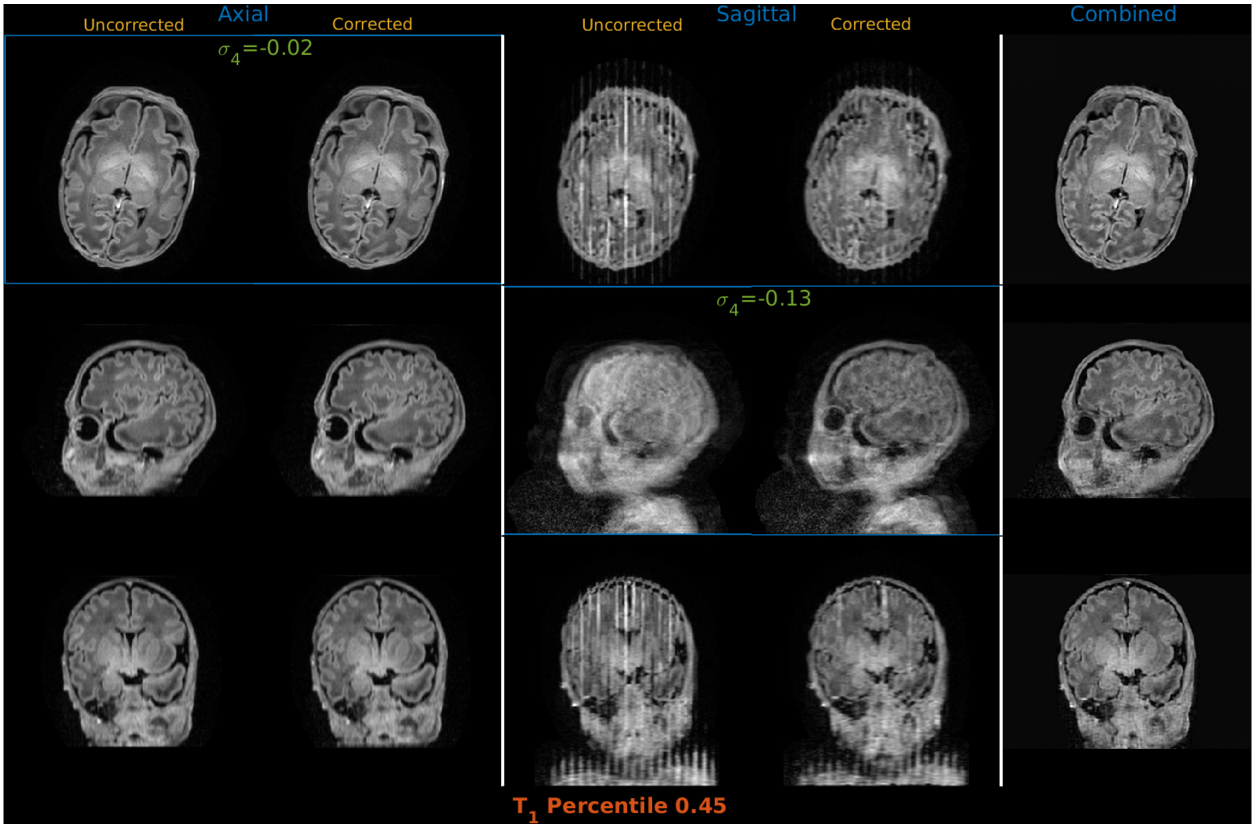

Supporting Figure S15

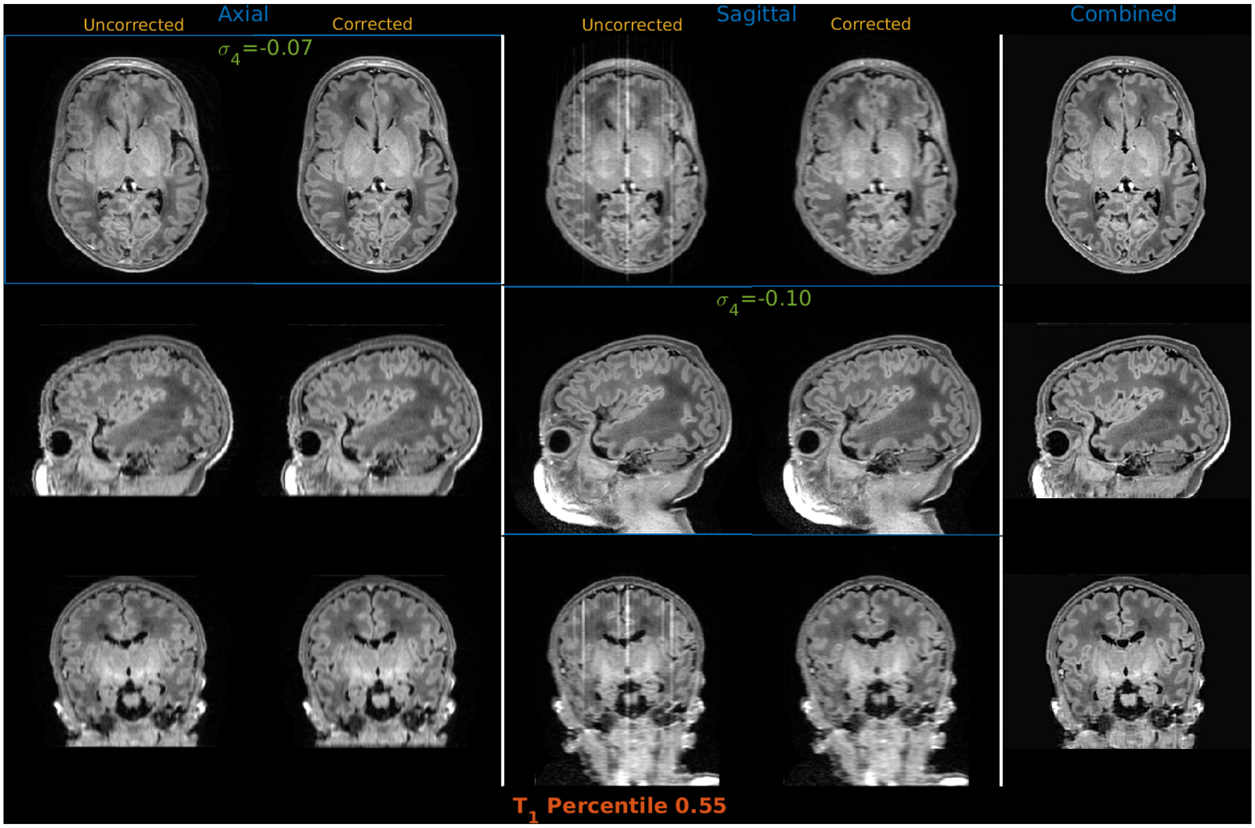

Supporting Figure S16

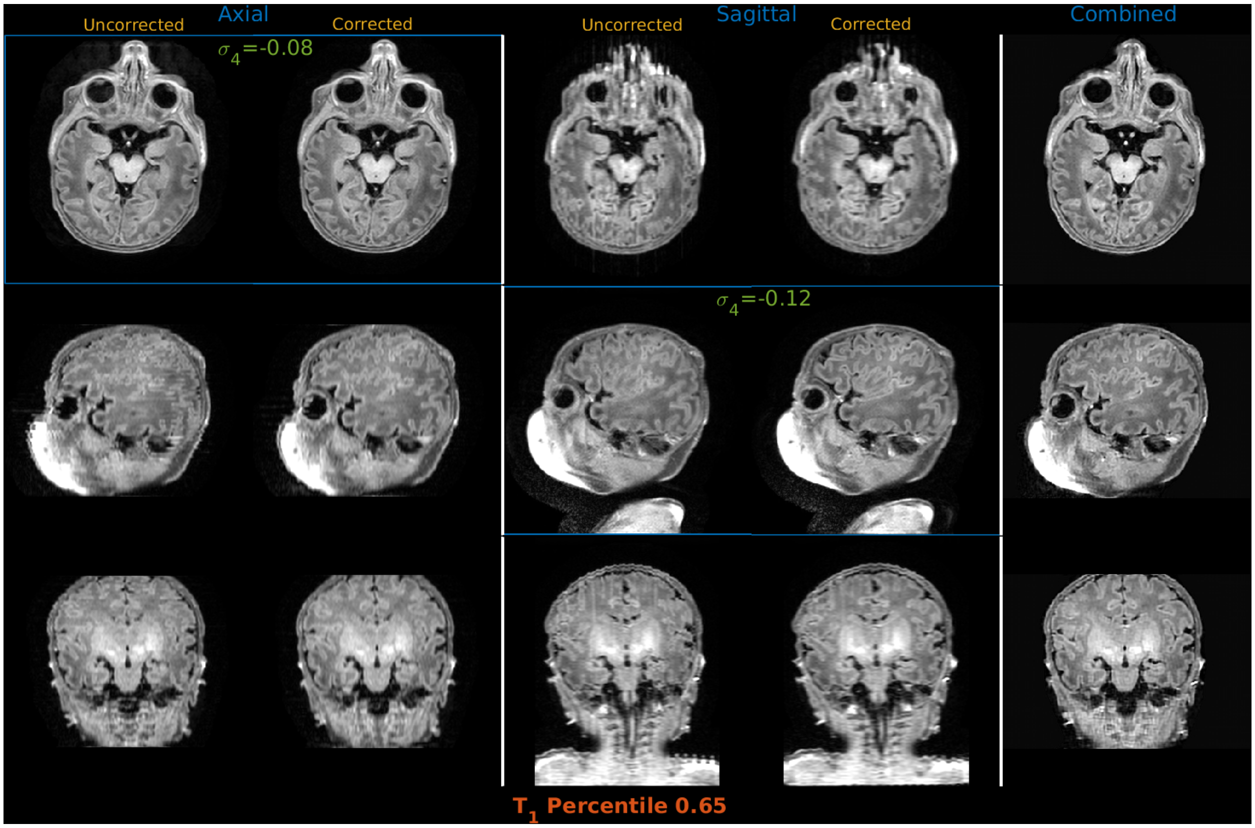

Supporting Figure S17

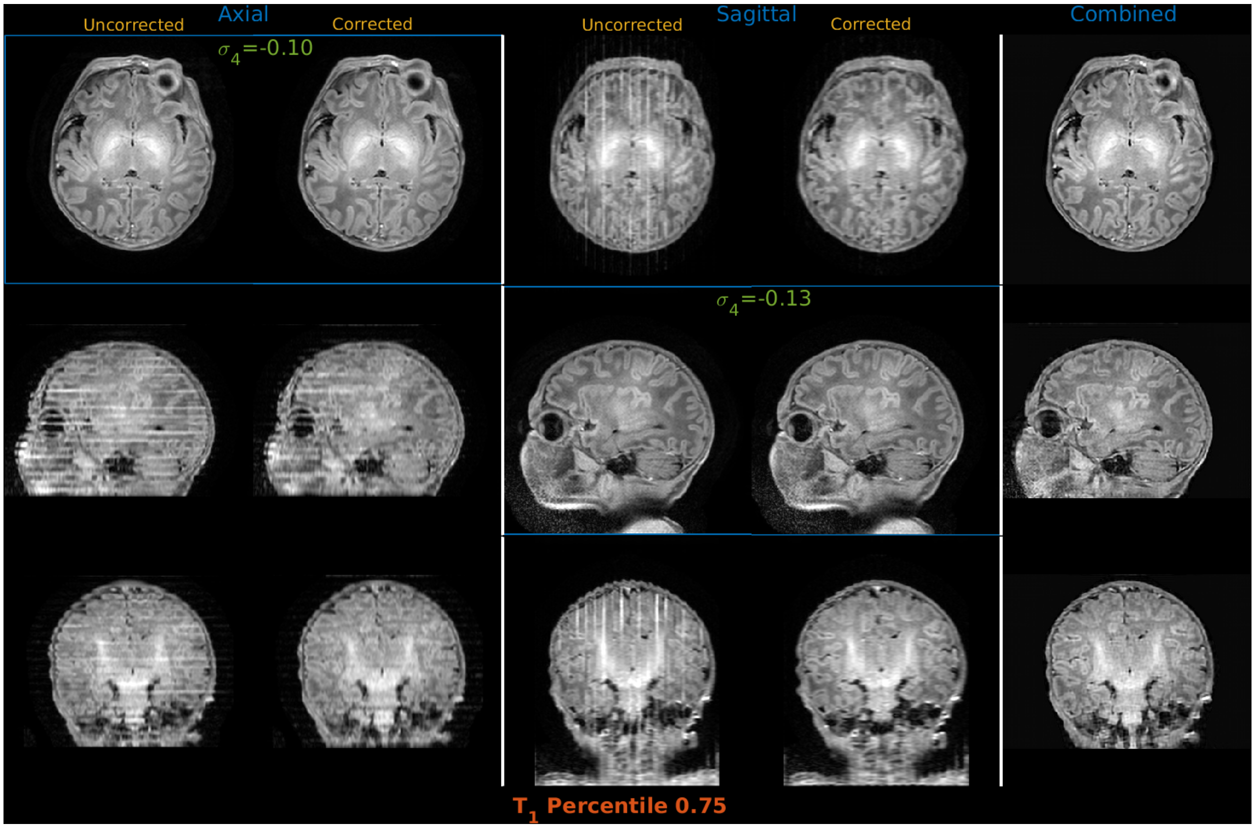

Supporting Figure S18

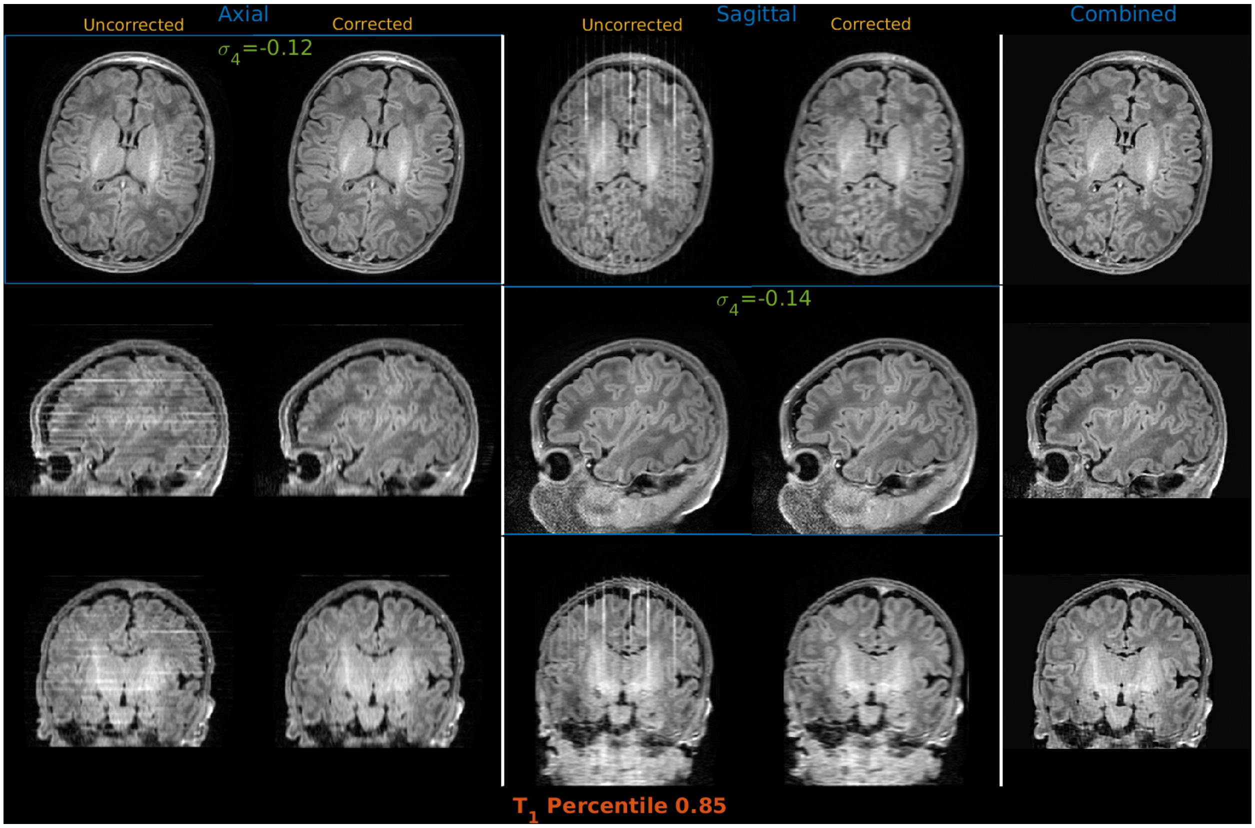

Supporting Figure S19

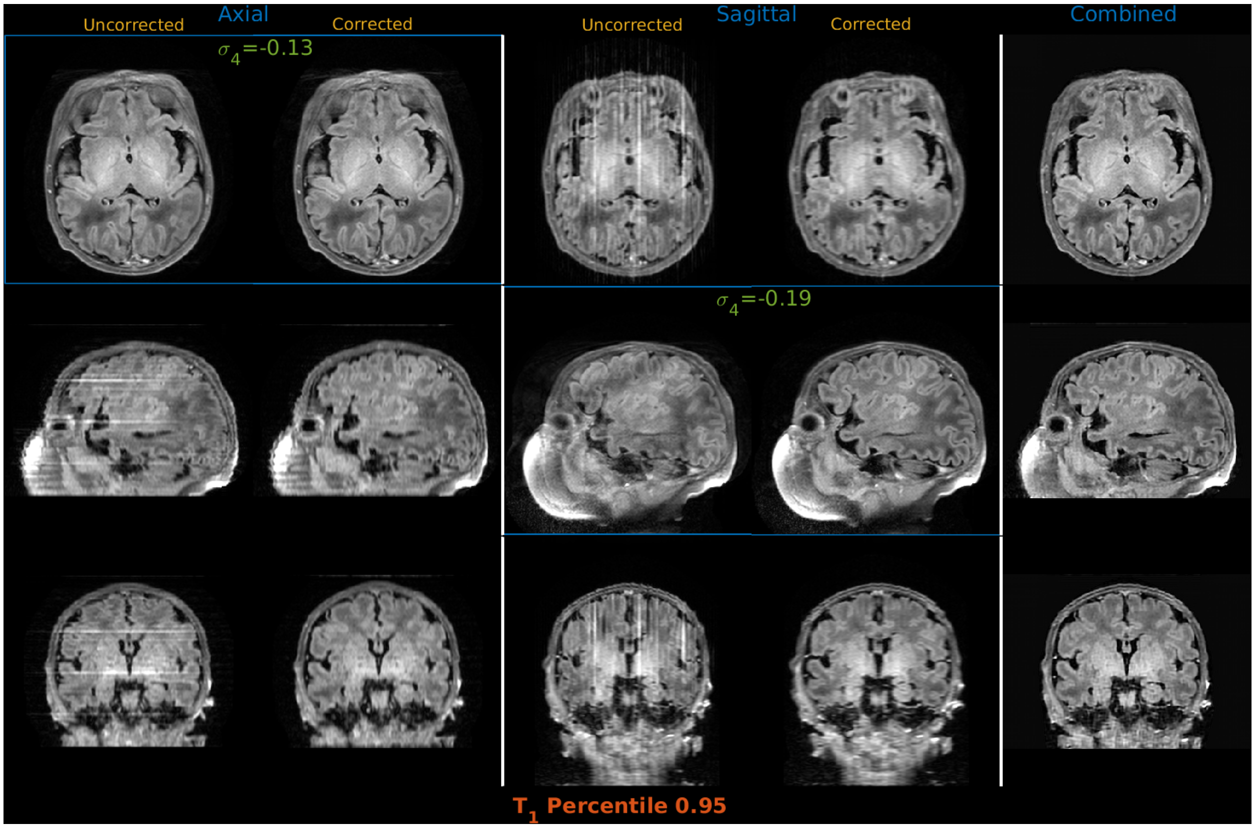

Supporting Figure S20
